# Copper on Calcium Carbonate and Marble Waste as Recyclable Catalysts for Ultrasound‐Assisted One‐Pot Synthesis of 1,4‐Disubstituted 1,2,3‐Triazoles

**DOI:** 10.1002/cssc.70944

**Published:** 2026-08-19

**Authors:** Riccardo Serra, Dipak J. Fartade, Pietro Caboni, Cristina Carucci, Andrea Salis, Enrica Tuveri, Antonella Rossi, Andrea Citarella, Andrea Porcheddu

**Affiliations:** ^1^ Department of Chemical and Geological Sciences University of Cagliari Cittadella Universitaria di Monserrato Monserrato Italy

**Keywords:** circular economy, click chemistry, green chemistry, marble powder, synthesis, triazoles, waste

## Abstract

A simple heterogeneous copper catalyst was prepared by adsorbing Cu(II) ions from an aqueous CuSO_4_ solution onto calcium carbonate (CaCO_3_) and marble waste microfine dust from the Carrara production area. The resulting materials (CaCO_3_–Cu and Carrara–Cu) were characterized by FTIR, PXRD, SEM–EDX, TGA, and zeta potential measurements, confirming copper uptake while preserving the carbonate framework. The catalytic activity of the materials was evaluated in the copper‐catalyzed azide–alkyne cycloaddition (CuAAC) reaction. Using glucose as a reducing agent, the reactions were performed in water under ultrasound irradiation at 30 °C, generating the active Cu(I) species in situ. A model three‐component reaction between benzyl bromide, sodium azide, and phenylacetylene afforded the corresponding 1,4‐disubstituted 1,2,3‐triazole in 81% isolated yield after 1 h. The scope of the methodology was evaluated across a range of substituted aryl alkynes and benzyl bromides as reaction partners, affording the corresponding triazoles in moderate to good yields. The catalyst was recovered by simple filtration and reused in subsequent reaction cycles. The use of water as the reaction medium, mild operating conditions, catalyst recyclability, and the successful application of marble waste as a catalyst support make this methodology a practical and sustainable approach for the synthesis of 1,2,3‐triazoles.

## Graphical abstract

A sustainable CaCO_3_‐supported copper catalyst, prepared by simple adsorption of Cu(II) onto natural and marble waste‐derived carbonate materials, promotes ultrasound‐assisted CuAAC reaction in water. The work provides straightforward access to 1,4‐disubstituted 1,2,3‐triazoles under mild conditions while combining catalyst recyclability with the valorization of mineral waste.

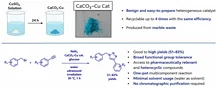

## 1 Introduction

1,2,3‐Triazoles display a broad spectrum of biological activities and have therefore become privileged motifs in medicinal chemistry [1, 2, 3]. Members of this class have been reported to exhibit antiviral [4, 5], anticancer [6, 7, 8], anticonvulsant [9], anti‐inflammatory [7], and antimicrobial activities [10, 11], among a wide range of other pharmacological applications. Their structural versatility enables them to act as bioisosteres for several functional groups, most notably amides, which underpins their widespread use in the design and optimization of bioactive molecules [1]. Among the available synthetic methods, the copper(I)‐catalyzed azide–alkyne cycloaddition (CuAAC) stands as a cornerstone of click chemistry, providing rapid and highly regioselective access to 1,4‐disubstituted 1,2,3‐triazoles with broad functional‐group tolerance and excellent atom economy (AE) [12, 13, 14]. Despite this remarkable synthetic efficiency, many established CuAAC protocols still rely on homogeneous copper salts, most commonly CuSO_4_ combined with an external reducing agent, and operating in mixed organic/aqueous media. Elevated temperatures are frequently required to overcome mass‐transfer limitations or solubility constraints. Taken together, these features complicate catalyst recovery, increase solvent demand, and raise concerns regarding copper leaching, issues that are particularly critical for applications in medicinal chemistry and materials science.

In response, recent efforts have increasingly focused on developing of greener and more environmentally benign CuAAC catalytic platforms [15, 16]. For example, Zhang et al. reported a biomimetic hybrid system in which Cu(II) is immobilized on a CaCO_3_/carboxymethylcellulose (CMC) matrix, enabling CuAAC reactions in water at 70 °C [17]. While effective, this approach relies on a biopolymeric templating agent and requires elevated temperatures to achieve high conversions. Along similar lines, Vilé and coworkers developed a single‐atom Cu catalyst supported on mesoporous carbon nitride, delivering excellent yields in *N*,*N*‐dimethylformamide (DMF) at 100–140 °C, albeit through a multistep catalyst synthesis and under solvent‐intensive conditions [18]. Bio‐inspired copper oxide nanoparticles derived from clove extract have also been reported as efficient CuAAC catalysts in DMF/water at 90 °C, affording yields of up to 98% [19].

Although these strategies improve catalyst recovery and reuse, they typically remain dependent on specialized supports, complex preparation protocols, organic cosolvents, and/or substantial thermal input.

Building on our ongoing efforts toward the sustainable synthesis of biologically relevant 1,2,3‐triazoles [20], we posit that meaningful gains in catalytic sustainability can emerge from the deliberate convergence of two simplifying elements: the use of abundant, intrinsically benign supports, ideally sourced from waste streams, and the execution of reactions in genuinely low‐impact media under mild conditions. Yet, a CuAAC platform that simultaneously integrates water as the sole reaction medium, ambient‐temperature operation, and mineral‐supported copper derived from abundant or waste carbonate sources within a circular‐economy framework remains conspicuously absent [21, 22, 23, 24].

Comprehensive characterization by Fourier‐transform infrared (FTIR) spectroscopy, powder X‐ray diffraction (PXRD), scanning electron microscopy coupled with energy‐dispersive X‐ray analysis (SEM–EDX), thermogravimetric analysis (TGA), and zeta potential measurements confirms efficient copper uptake while preserving the carbonate framework, with calcite remaining the dominant phase. These findings are consistent with the formation of surface‐anchored copper species rather than bulk conversion into a discrete copper salt.

The resulting copper‐loaded marble materials directly enable a practical catalytic platform for aqueous CuAAC. The surface‐immobilized copper is readily activated under mild sonication, allowing the click transformation to proceed efficiently in distilled water at 30 °C. Owing to the heterogeneous nature of the system, work‐up is inherently straightforward, with the catalyst being recovered by simple filtration and reused with minimal handling. From both application and sustainability perspectives, Carrara marble, specifically scraps, functions not merely as an inert support but as a value‐generating, waste‐derived scaffold that converts an industrial byproduct into a functional catalytic material [25]. When combined with a water‐only, low‐temperature operating, this approach offers a tangible reduction in solvent demand and energy input while preserving the operational simplicity required for routine synthetic application.

## 2 Results and Discussion

### 2.1 Catalyst Preparation and Characterization

To operationalize the circular‐economy premise of this study, a catalyst preparation strategy that matched the simplicity and low environmental footprint of the targeted aqueous CuAAC protocol was required. Accordingly, a straightforward “adsorb‐and‐use” strategy was adopted, enabling direct copper immobilization from water onto the calcium carbonate surface at ambient temperature, with metal loading providing a direct link between catalyst preparation, performance, and reusability.

**FIGURE 1:**
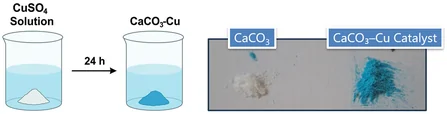
Scheme of CaCO_3_–Cu catalyst preparation.

Quantifying copper uptake at this stage is therefore essential. The CaCO_3_–Cu catalyst was prepared via spontaneous adsorption of Cu(II) from aqueous CuSO_4_ onto CaCO_3_ powder. In a representative procedure, CaCO_3_ was placed in contact with a 0.02 M CuSO_4_ solution and stirred for 24 h at room temperature (Figure 1). After this, the suspension was then filtered, and the residual copper concentration in the filtrate was quantified by UV–visible spectroscopy. Mass‐balance analysis revealed a copper loading of approximately 1.32 mmol g^−1^ (≈ 0.132 mmol per 100 mg, corresponding to ∼8.4 wt% Cu).

**FIGURE 2:**
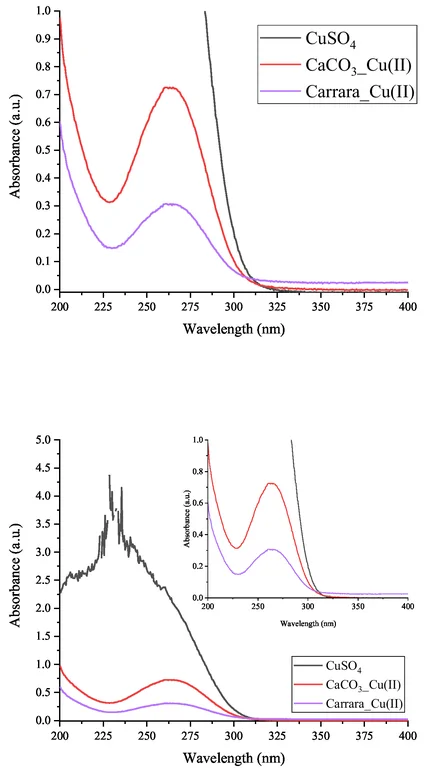
UV–visible absorption spectra of the initial CuSO_4_ solution (black curve) and those recorded after contact with different carbonate materials (commercial CaCO_3_ curve in red, Carrara marble powder in violet).

Figure 2 compares the UV–visible spectrum of the initial CuSO_4_ solution with those recorded after 24 h of contact with the different carbonate supports. The characteristic CuSO_4_ absorption band at ca. 264 nm, assigned to ligand‐to‐metal charge‐transfer transitions of hydrated Cu(II) species, decreases markedly after exposure to CaCO_3_, indicating substantial removal of copper from the aqueous phase (Figure 2, red trace).

In line with the circular‐economy rationale of this study, the same “contact‐and‐adsorb” protocol was applied to a relevant carbonate substrate, Carrara marble, affording the corresponding copper‐loaded material (Carrara–Cu). Notably, UV–visible analysis of the post‐contact solutions revealed an even more pronounced attenuation of the Cu(II) absorption bands after treatment with Carrara marble compared with commercial CaCO_3_ (Figure 2, violet trace), indicating highly efficient copper uptake by the natural material. Overall, the progressive disappearance of the characteristic Cu(II) features in solution provides direct evidence for the spontaneous immobilization of copper onto the carbonate matrix, most likely through surface coordination to carbonate and/or oxide sites. This adsorption step affords a readily accessible Cu‐loaded carbonate solid, which can be directly employed as an active and recyclable heterogeneous catalyst for aqueous CuAAC under mild sonochemical conditions.

In the current system, the UV–visible spectra exhibited a reduction in the Cu(II) absorption bands, with no new phase signatures appearing in the PXRD. This suggests that surface adsorption predominates over the bulk conversion to copper carbonate. The blue color of the recovered solid is consistent with predominant surface‐associated Cu species, possibly with minor local precipitation of basic copper carbonate phases.

Table 1 summarizes the UV–vis‐derived Cu(II) concentration remaining in solution after 24 h of contact with the carbonate supports. The diagnostic Cu(II) absorption band (*λ*_max_ ≈ 264 nm) decreased substantially after exposure to CaCO_3_ and even more after contact with Carrara marble, consistent with efficient copper uptake (Figure S1). Together with the light‐blue coloration of the recovered solids, these data support spontaneous immobilization of Cu onto the carbonate matrix (Figure 2).

**TABLE 1:** UV–visible absorption parameters for CuSO_4_‐ and Cu‐loaded carbonate samples.

	***A*,** **a.u.**	***λ***, **nm**	***C*, M**
CuSO_4_	>3.00	—	0.0200 ± 0.0003
CaCO_3_–Cu	0.723 ± 0.008	264.0 ± 0.3	0.0068 ± 0.0001
Carrara–Cu	0.306 ± 0.008	264.0 ± 0.3	0.0029 ± 0.0007

To investigate the relationship between the adsorption‐based preparation method and the catalytic behavior observed in water, it is essential to establish (i) whether the carbonate framework is retained after copper uptake and (ii) how copper is anchored to the solid (surface‐bound vs. formation of a separate crystalline phase). Accordingly, we characterized the pristine supports (commercial CaCO_3_ and Carrara marble) and the corresponding Cu‐loaded materials (CaCO_3_–Cu and Carrara–Cu) using FTIR spectroscopy, PXRD, SEM–EDX, and TGA. In addition, the zeta potential (ζ) and dispersion pH were measured before and after copper adsorption and after the click reaction.

**FIGURE 3:**
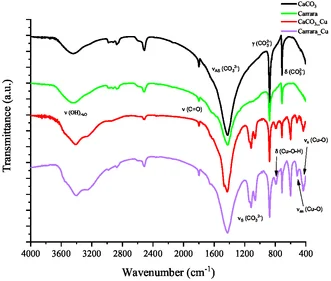
FTIR spectra of CaCO_3_ (black), Carrara marble (green), CaCO_3_–Cu catalyst (red), and Carrara–Cu catalyst (violet).

FTIR spectroscopy was first employed to compare pristine CaCO_3_ and Carrara marble with their Cu‐loaded counterparts (CaCO_3_–Cu and Carrara–Cu). As shown in Figure 3 (commercial CaCO_3_, black line of the plot; Carrara marble, green; CaCO_3_–Cu, red; Carrara–Cu, violet), all samples displayed the diagnostic vibrations of calcite at 1405 cm^−1^ (ν_AS_, asymmetric stretching of CO_3_^2^^−^), 874 cm^−1^ (γ, out‐of‐plane bending), and 712 cm^−1^ (δ, in‐plane bending), confirming that the carbonate framework was preserved upon copper uptake.

In the Cu‐loaded materials, additional weak features emerged at 1140, 1117, and 1063 cm^−1^, together with a signal around 600 cm^−1^, which were consistent with Cu–O vibrational modes (symmetric/antisymmetric stretching). Importantly, the spectra do not show intense absorptions typically associated with crystalline CuO, suggesting that copper is not present as a separate oxide phase but rather as a surface‐bound species that interacts with the carbonate matrix. Overall, the FTIR data support a picture in which carbonate integrity is retained while copper is anchored at the surface through Cu–O linkages, consistent with the formation of a robust heterogeneous catalyst platform.

**FIGURE 4:**
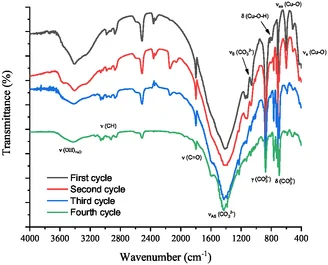
FTIR spectra of CaCO_3_–Cu catalyst recovered after successive catalytic cycles.

Figure 4 presents the FTIR spectra of the CaCO_3_–Cu catalyst before and after its catalytic application. The catalyst was readily recovered by simple filtration. A detailed investigation of catalyst recovery and reuse is presented in Section 2.3. The characteristic carbonate vibrational bands at 1405, 872, and 713 cm^−1^ remained evident, confirming the preservation of the calcite framework during the catalysis process. Minor alterations in the ν(CO_3_^2−^) asymmetric stretching band intensity and a slight broadening in the O–H region (3500–3200 cm^−1^) suggest weak surface coordination of copper and the presence of adsorbed water molecules. Additionally, a band around 2100 cm^−1^ is attributed to firmly bound water species. A faint signal near 620 cm^−1^, likely corresponding to Cu–O stretching vibrations, indicates the persistence of the surface‐bound copper species. Overall, the absence of new absorptions or significant shifts suggests that the catalyst maintained its structural integrity, with copper remaining on the carbonate surface without being converted into separate copper oxide or basic copper carbonate phases. These findings confirm the robustness and recyclability of the CaCO_3_–Cu catalyst under aqueous and ultrasound‐assisted conditions.

**FIGURE 5:**
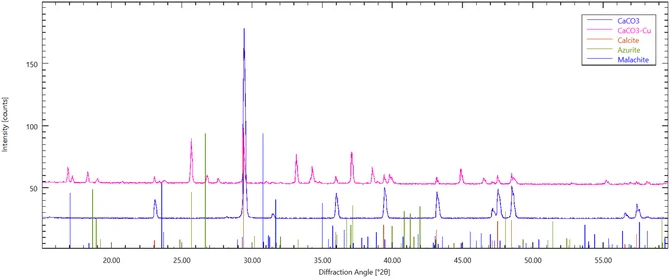
PXRD patterns of the catalysts.

PXRD was employed to probe possible phase changes associated with copper uptake. Figure 5 compares the diffraction pattern of pristine CaCO_3_ (blue) with that of the Cu‐loaded material (CaCO_3_–Cu, red). In both cases, the characteristic reflections of calcite are preserved, indicating that the carbonate framework remains the dominant crystalline phase after exposure to the CuSO_4_ solution. In the Cu‐loaded sample, a limited number of weak additional reflections can be discerned, which may tentatively be associated with basic copper carbonate polymorphs (e.g., azurite or malachite). However, the low intensity and poor definition of these features, likely arising from limited crystallinity and sample heterogeneity, preclude unambiguous phase assignment. Accordingly, while these faint signals may suggest partial surface precipitation of copper carbonate species, the overall diffraction pattern confirms that bulk calcite is retained. Taken together with the FTIR data, these results support a picture of a carbonate matrix decorated with surface‐associated copper species, rather than extensive conversion into a separate crystalline copper salt phase.

SEM images together with the corresponding EDX analysis (Figure S2) show CaCO_3_ particles with a well‐defined faceted morphology, which is largely preserved after copper loading. While only trace amounts of copper were detected for pristine CaCO_3_ (0.3 wt%), a substantially higher copper content was observed for CaCO_3_–Cu (17.3 wt%; Figure S2), confirming efficient surface enrichment upon adsorption [26]. The higher wt% detected by SEM–EDX likely reflects localized surface copper adsorption rather than the average bulk loading determined by UV–vis.

Zeta potential measurements of bare commercial CaCO_3_ and Carrara marble are in good agreement with literature values and vary as a function of dispersion pH (Table 2) [27]. Following copper adsorption, a decrease in the absolute zeta potential values is observed, plausibly reflecting charge screening effects associated with surface‐bound copper species. After the CuAAC reaction, the catalyst exhibits a strongly negative zeta potential (–37 ± 1 mV), which may be attributed to partial removal of adsorbed copper species and/or to the presence of negatively charged surface functionalities introduced during catalysis, such as gluconate residues [28].

**TABLE 2:** Zeta potential (*ζ*) and dispersion pH of the catalysts before and after Cu(II) adsorption and click chemistry, measured in Milli‐Q water.

Sample	pH	*ζ*, mV
CaCO_3_	8.3	−19 ± 3
CaCO_3_ Carrara	9.4	−17 ± 3
CaCO_3_–Cu	8.0	−4 ± 2
CaCO_3_–Cu Carrara	8.9	−6 ± 3
CaCO_3_–Cu‐post‐click^a^	7.9	−37 ± 1
CaCO_3_–Cu‐post‐click^b^	7.8	−9 ± 2

a Refers to entry 5 in Table 3.

b Refers to entry 6 in Table 3.

**FIGURE 6:**
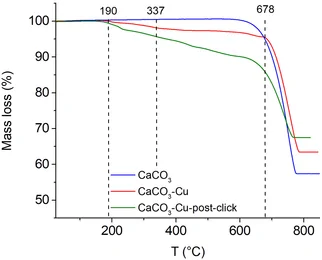
Thermogravimetric analysis (TGA) from 25 to 850 °C of CaCO_3_ (blue curve), CaCO_3_–Cu (red curve), and CaCO_3_–Cu‐post‐click (green curve).

Thermogravimetric analysis (TGA) showed negligible mass loss for all samples between 25 and 190 °C, confirming the absence of residual water or solvent. In the range of 190–337 °C, CaCO_3_‐Cu showed a higher mass loss than pure CaCO_3_ (Figure 6). This mass loss is consistent with the thermal degradation of Cu(II) adsorption complexes formed upon Cu loading onto CaCO_3_ [29, 30]. Within the same temperature range, CaCO_3_–Cu‐post‐click (refer to entry 5 in Table 3) exhibited a loss of 3.6% and a significantly higher mass loss over the range 337–678 °C, with the latter likely due to the presence and thermal degradation of organic moieties introduced by the post‐click modification. Finally, the mass loss measured above 678 °C was assigned to the decomposition of the carbonate framework [31]. Data are reported in Table 4.

**TABLE 3:** Optimization of the reaction conditions.^a^

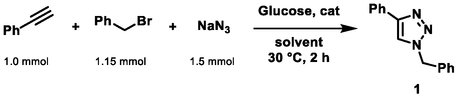				
Entry	Solvent	Catalyst	Time	**Yield** b
1	DMF/water^c^	CuSO_4_	2 h	82%
2	DMF/water^c^	CaCO_3_–Cu	2 h	62%
3	Water	CaCO_3_–Cu	2 h	86%
4	Water	CaCO_3_–Cu^d^, ^e^	Overnight	85%
5^f^	Water	CaCO_3_–Cu	1 h	81%
6^f^	Water	CaCO_3_–Cu^g^	1 h	65%
7^f^	Water	Carrara–Cu	1 h	70%

a The reactions were performed using phenylacetylene (1.0 mmol), benzyl bromide (1.15 mmol), sodium azide (1.5 mmol), glucose (0.2 mmol), and the catalyst (40 mg) in a 50 mL flask using 5 mL of solvent at 30 °C for 2 h.

b Isolated yields.

c Solvent ratio 3:1.

d The reaction was performed with the recycled catalyst from entry **3.**

e The reaction occurred overnight.

f Reaction performed in a glass vial using water (0.8 mL, *η* = 2) at 30 °C for 1 h under ultrasound irradiation.

g The reaction was performed with the recycled catalyst from entry **5.**

**TABLE 4:** Mass loss (%) from TGA measurements related to the main decomposition.

**Sample**	Δ*m*, %		
	**25–190, °C**	**190–337, °C**	**337–678, °C**
CaCO_3_	0.3	0.2	5.5
CaCO_3_–Cu	0.02	1.8	2.7
CaCO_3_–Cu–post‐click^a^	0.6	3.6	9.7

a Refers to entry 5 in Table 3.

### 2.2 Application of the CaCO_3_–Cu Catalyst for the Synthesis of 1,2,3–Triazoles

To frame the catalytic evaluation of the carbonate‐supported materials, we next turned to the CuAAC reaction under conditions that reflect both established practices and the sustainability targets of this work. In particular, CuAAC is commonly implemented using a stable Cu(II) precursor and generating the active Cu(I) species in situ with a mild reductant, which also helps to limit the side reactions associated with direct Cu(I) sources. With this in mind, we selected a representative one‐pot three‐component click protocol as a benchmark. A combination of phenylacetylene, benzyl bromide, and sodium azide in the presence of glucose was selected for the optimization of the reaction parameters and the assessment of the activity of the newly prepared heterogeneous catalysts (entry 1 in Table 3). Glucose was employed as a mild and environmentally benign reducing agent to generate the catalytically active Cu(I) species in situ from the supported Cu(II) precursor. Under these conditions, the reaction afforded the desired product in 62% yield after 2 h at 30 °C, confirming the feasibility of the process with our catalyst (entry 2 in Table 3). Once the reaction was complete, as monitored by TLC, the catalyst was easily recovered through simple filtration, washed with ethyl acetate, and the filtrate underwent a conventional extractive work‐up. Notably, the final crude mixture contained only the desired product, with a purity exceeding 95%, thereby avoiding chromatographic purification and reducing solvent use and waste generation. To enhance the sustainability of the process, the reaction was repeated under solvent‐minimized and environmentally friendly conditions by eliminating the organic co‐solvent. The same reaction parameters were applied (entry 3 in Table 3), using water as the sole reaction medium (5 mL). Under these conditions, the product yield improved to 86%, demonstrating that the transformation can be efficiently conducted in water alone, in accordance with green chemistry principles. Moreover, the catalyst was easily recovered and reused in a subsequent catalytic cycle. In entry 4 (Table 3), the reaction was conducted using the catalyst recovered from entry 3, yielding a comparable yield of 85%. These results show the robustness and recyclability of the catalyst, thereby contributing to waste reduction and improved resource efficiency. Finally, to further decrease the solvent usage and reaction time while maintaining mild reaction conditions, the reaction was explored under ultrasonic irradiation [32]. The reaction (entry 5 in Table 3), conducted in a glass vial with a minimal solvent volume (0.8 mL, *η* = 2), was completed in just 1 h at 30 °C, yielding the product in 81% yield. These findings identified the ultrasonic‐assisted protocol as the optimal and most sustainable set of reaction conditions for further process development and exploration of the reaction scope. The catalyst used in entry 5 was recovered, and in this case as well the recovery percentage was approximately 60%. The reaction was repeated under the same conditions using the catalyst recovered from entry 5 (entry 6 in Table 3): after 1 h at 30 °C, the reaction afforded a 65% yield, demonstrating that the ultrasound‐assisted process also proceeds with the recycled catalyst, which remained catalytically active upon reuse. Subsequently, Carrara marble powder functionalized with CuSO_4_ (Carrara–Cu) was tested under ultrasonic conditions (entry 7 in Table 3). Notably, this natural carbonate‐based catalyst facilitated the formation of the desired triazole with an isolated yield of 70% after only 1 h, using the same sonication‐assisted protocol applied to CaCO_3_–Cu. This outcome confirms that marble‐derived supports can serve as efficient and sustainable catalytic platforms for aqueous CuAAC reactions, albeit with slightly lower performance than that of commercial calcium carbonate (CaCO_3_).

**FIGURE 7:**
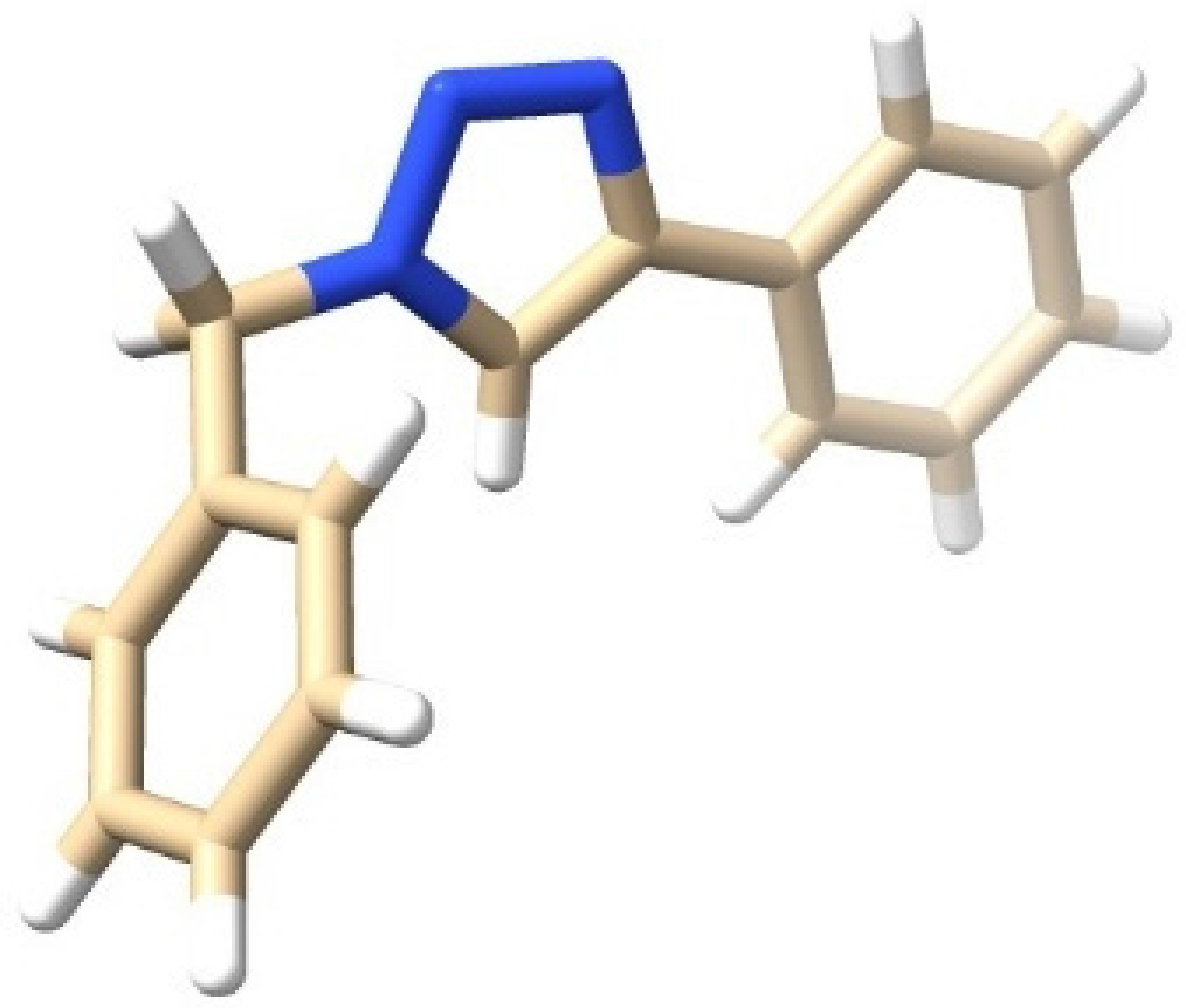
Single‐crystal X‐ray structure of compound **1** (1‐benzyl‐4‐phenyl‐1*H*‐1,2,3‐triazole), confirming the expected 1,4‐regiochemistry typical of CuAAC reactions. The crystal belongs to the space group *P2_1_/c* with unit cell parameters: *a* = 6.0070(5) Å, *b* = 8.0455(7) Å, *c* = 25.344(2) Å, β = 94.122(2)° (CCDC refcode NILLEZ01, deposition number 231980).

The target compound (**1**) was successfully crystallized, and its structure was determined using single‐crystal X‐ray diffraction (Figure 7). The analysis confirmed that the molecule corresponded to 1‐benzyl‐4‐phenyl‐1*H*‐1,2,3‐triazole (CCDC refcode NILLEZ01, deposition number 231980), exhibiting the characteristic 1,4‐disubstitution pattern typical of CuAAC reactions. The unit cell parameters (space group: *P*2_1_/*c*, cell: *a* 6.0070(5) Å, *b* 8.0455(7) Å, *c* 25.344(2) Å, *α *= 90° *β *= 94.122(2)°, *γ = *90°) match those reported in the Cambridge Structural Database, confirming the expected geometry and connectivity of the click product [33].

To assess the efficiency of the CaCO_3_–Cu catalyst, its performance was compared with that of Cu‐based heterogeneous systems reported in previous studies. CuO nanoparticles synthesized from natural extracts, such as those derived from clove, demonstrate high CuAAC activity in DMF/H_2_O (8:2) at 90 °C, achieving nearly quantitative yields [19]. Similarly, a biomimetic hybrid catalyst, with Cu^2+^ immobilized on a CaCO_3_/CMC matrix, has been shown to facilitate CuAAC reactions in water at 70 °C, achieving excellent conversions but necessitating a biopolymeric templating agent and elevated temperatures [17]. In contrast, the current CaCO_3_–Cu system operates under much milder conditions, utilizing pure water at 30 °C, with the assistance of ultrasound, without the need for organic cosolvents or polymeric supports, while still maintaining competitive yields and catalyst recyclability. The CaCO_3_–Cu catalyst achieved high conversion in water within 1 h at 30 °C, delivering competitive yields while reducing energy input and solvent use.

Additionally, the ultrasound‐assisted protocol reduces reaction time and minimizes solvent usage, thereby enhancing the environmental friendliness and sustainability of the process. The use of calcium carbonate as a natural, inexpensive, and readily available support further sets this system apart. Unlike nanoparticle‐based materials that require precursor synthesis or stabilizing agents, Cu adsorption onto CaCO_3_ occurs spontaneously and substantially, as confirmed by UV–visible monitoring of the CuSO_4_ solution after 24 h of contact. The resulting CaCO_3_–Cu solid represents a low‐cost and environmentally benign catalytic platform.

**SCHEME 1:**
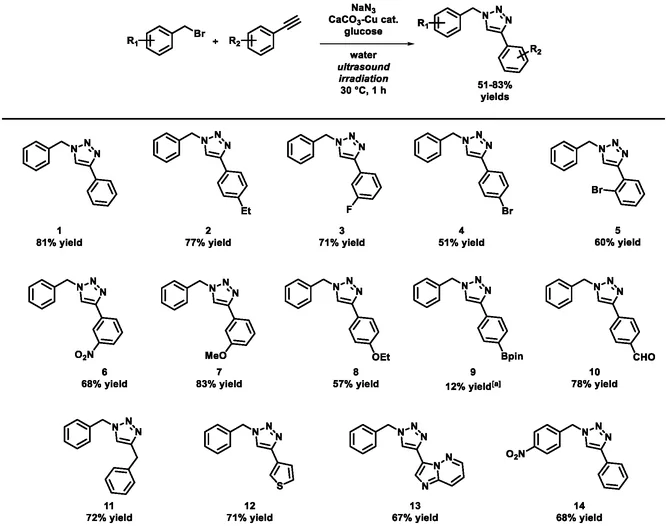
Reaction scope. The reaction was performed using phenylacetylene (1.0 mmol), benzyl bromide (1.15 mmol), sodium azide (1.5 mmol), glucose (0.2 mmol), and the catalyst (40 mg) in a glass vial using water (*η* = 2) as the solvent at 30 °C for 1 h under ultrasound‐assisted conditions. Except for **9**, all products were isolated in pure form after simple ethyl acetate extraction, avoiding column chromatography and reducing silica and solvent consumption. [a] Yield determined by ^1^H NMR analysis of the crude mixture.

To investigate the substrate scope, we employed the optimized ultrasound‐assisted conditions (entry 5 in Table 3) to synthesize some 1,2,3‐triazoles, to demonstrate the broad applicability of our method (Scheme 1). Under these conditions, the reaction was conducted on a 1.0 mmol scale using variously substituted phenylacetylenes and the CaCO_3_–Cu catalyst in a vial with minimal water (*η* = 2) under ultrasonic irradiation. This protocol enabled phenylacetylene to yield the corresponding triazole in 81% yield (**1**), confirming the method’s efficiency under mild and sustainable conditions. The 4‐ethyl derivative reacted smoothly, yielding **2** in 77% yield. Halogen‐substituted phenylacetylenes exhibited good reactivity, with yields ranging from 71% for the *meta*‐fluoro compound (**3**) to 51% for *para*‐bromo (**4**) and 60% for *ortho*‐bromo (**5**) compounds. The *meta*‐nitro derivative, with its strong electron‐withdrawing group, also reacted efficiently, yielding **6** in 68% yield. Electron‐donating substituents generally enhanced the reaction efficiency: *meta*‐methoxy (**7**) yielded 83% while para‐ethoxy (**8**) yielded 57%. Notably, the phenylacetylene derivative with a *para*‐boronic ester (**9**) showed negligible conversion (12%), as indicated by the crude NMR analysis. Additionally, *para*‐ethynylbenzaldehyde reacted well, yielding **10** in 78% yield. Substituted benzyl bromides were also tested; for instance, *para*‐nitrobenzyl bromide yielded product **14** with 68% yield. Except for **9**, all the final products were isolated in pure form after simple extraction with ethyl acetate, eliminating the need for column chromatography purification. This procedure eliminated the need for column chromatography and silica, thereby significantly reducing the environmental impact. Overall, these results highlight the broad applicability of the CaCO_3_–Cu catalyst to diverse substrates, with performance trends consistent with the substituent electronic effects.

### 2.3 Catalyst Recycling Studies and Proposed Mechanisms

**FIGURE 8:**
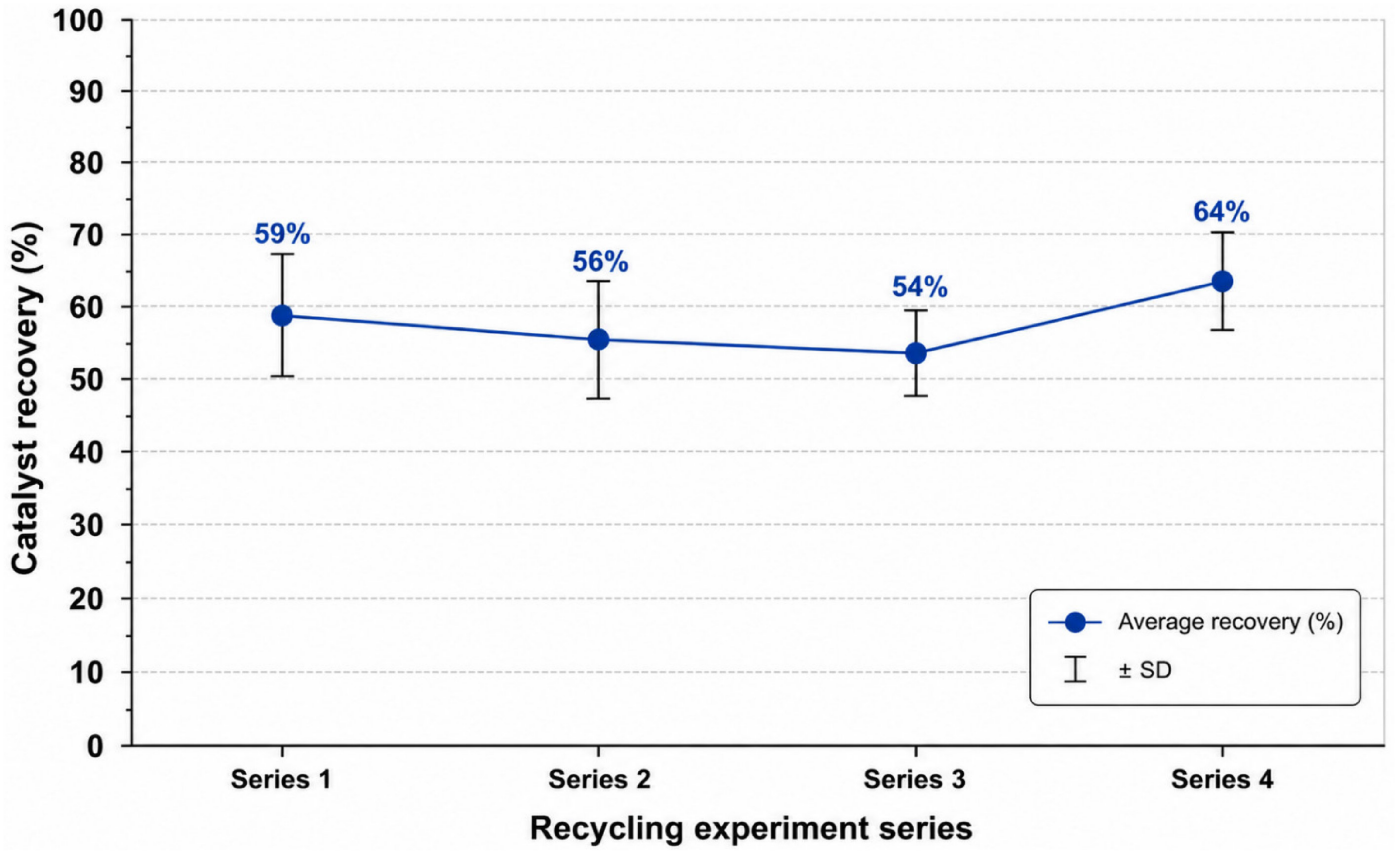
Recycling experiments.

To further evaluate catalyst recyclability, a series of recovery and reuse experiments were performed under the optimized ultrasound‐assisted CuAAC conditions (Figure 8). After each reaction cycle, the CaCO_3_–Cu catalyst was recovered by simple filtration, washed with ethyl acetate, dried, weighed, and then directly reused in subsequent reactions without any additional treatment. Across four independent recycling series, catalyst recovery ranged from 54% to 64%, with average recovery values of 59% ± 8%, 56% ± 9%, 54% ± 6%, and 64%, respectively (Figure 8 and Table S1). The partial loss of material is mainly attributed to mechanical losses during filtration, washing, and handling of the finely divided carbonate powder rather than to catalyst decomposition. The recovered material retained its catalytic activity over successive cycles, affording the corresponding triazole **1** in a consistent good yield. These results are consistent with the retention of catalytically competent copper species on the carbonate support.

**FIGURE 9:**
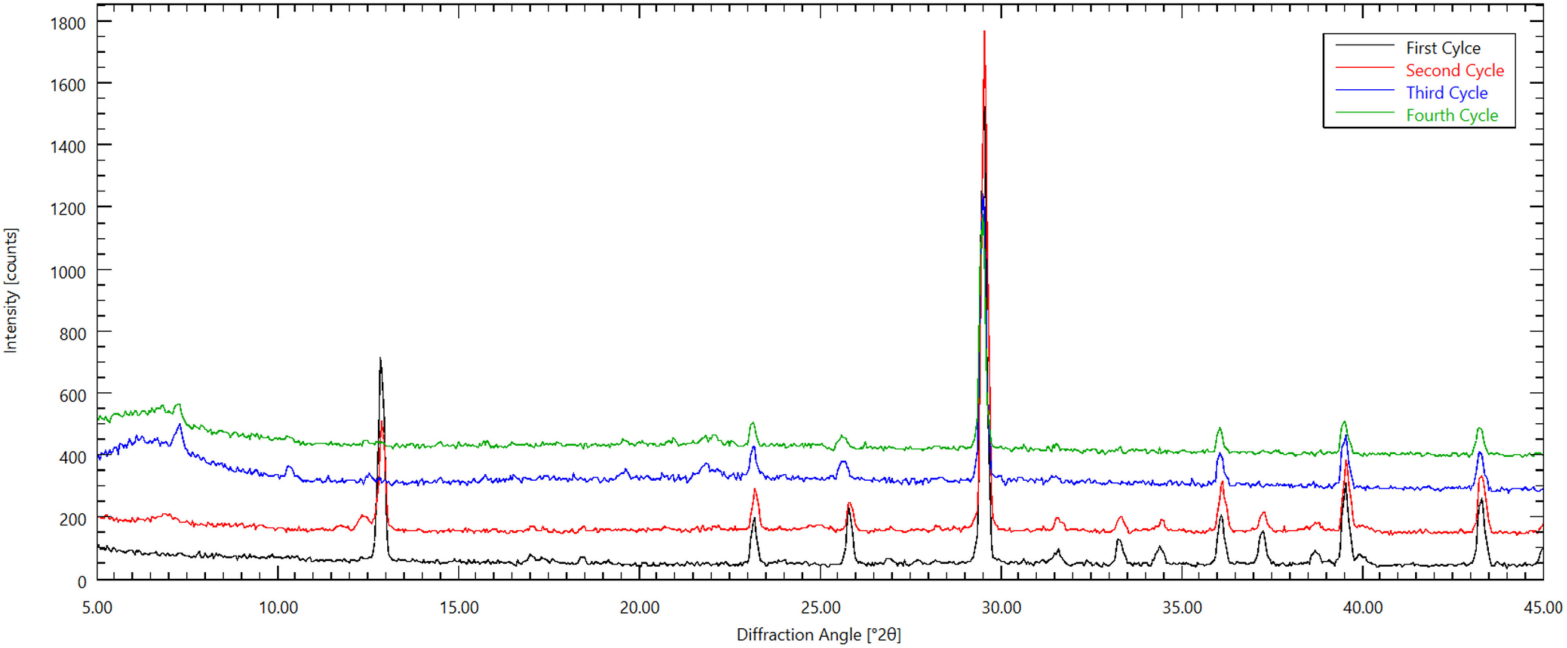
PXRD of the catalyst samples after one (black), two (red), three (blue), and four (green) reuse cycles.

To gain further insight into catalyst stability upon reuse, the recovered CaCO_3_–Cu catalyst was analyzed by PXRD after successive catalytic cycles (Figure 9). Diffraction patterns revealed that the characteristic reflections of calcite remained unchanged throughout the recycling experiments, indicating that the calcium carbonate support preserved its crystalline structure under the reaction conditions. The PXRD patterns showed a gradual attenuation of the low‐angle reflections associated with the freshly prepared Cu‐loaded material; the recovered material exhibited a profile with more amorphous characteristics, suggesting a progressive restructuring of the copper‐containing phase during catalyst reuse.

**SCHEME 2:**
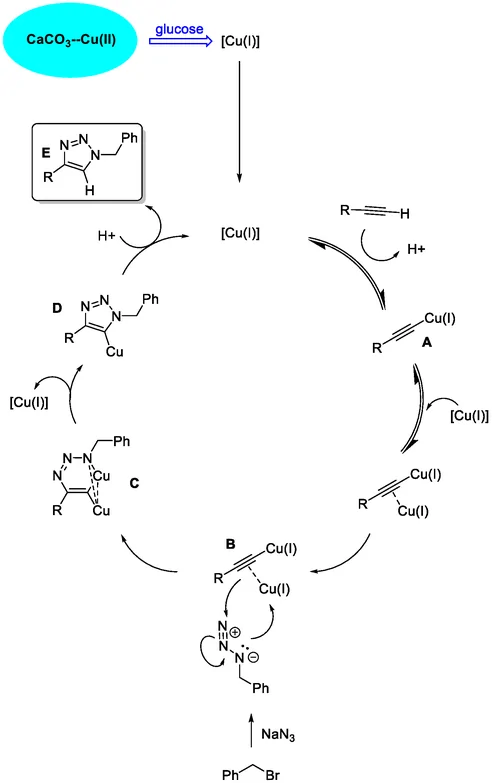
Proposed mechanism for the CuAAC reaction catalyzed by the CaCO_3_‐supported Cu catalyst. Surface Cu(II) species are reduced in situ by glucose to generate the catalytically active Cu(I) species, which follows the classic CuAAC catalytic cycle.

A plausible catalytic cycle is proposed based on the well‐established CuAAC mechanism reported by Fokin and collaborators (Scheme 2) [34]. Under the reaction conditions, glucose acts as a mild reducing agent, converting the surface‐bound Cu(II) species into catalytically active Cu(I). The resulting Cu(I) species enters the conventional CuAAC catalytic cycle through formation of a copper acetylide (A), followed by azide coordination (B), metallacycle formation (C), triazolide generation (D), protonation, and finally regeneration of the Cu(I) catalyst (E). Although no direct mechanistic investigations were performed, this proposal is consistent with the requirement of in situ Cu(I) generation in CuAAC reactions and with the absence of detectable Glaser coupling byproducts under the adopted reaction conditions.

### 2.4 Green Metrics Evaluation

To evaluate the sustainability of the heterogeneous CuAAC process, we examined key green chemistry metrics under representative conditions (Table 5). The theoretical AE was calculated from the ideal stoichiometry of benzyl bromide, sodium azide, and phenylacetylene forming the triazole product and inorganic NaBr byproduct (glucose excluded).

**TABLE 5:** Green metrics for selected conditions (glucose excluded).

Entry	Solvent	Yield, %	AE, %	RME, %	sPMI	E‐factor
**1**	DMF/water	82	69.6	48.68	2.05	1.05
**2**	Water	86	69.6	51.05	1.96	0.96
**3**	Water (US)	81	69.6	48.0	2.08	1.08
**4**	Water (US)	70	69.6	41.55	2.41	1.41

*N* *ote:* Solvent and catalyst excluded; stoichiometry: benzyl bromide 1.15 mmol, NaN_3_ 1.5 mmol, phenylacetylene 1 mmol; product MW = 235.28 g/mol.

Abbreviation: US, ultrasound.

$$\mathrm{AE}\;(\%)=({\mathrm{MW}}_{\text{\_}}\mathrm{product}/{\mathrm{\Sigma MW}}_{\text{\_}}\mathrm{reactants})\times 100$$

$$C_{7}H_{7}\mathrm{Br}+{\mathrm{NaN}}_{3}+C_{8}H_{6}\to C_{15}H_{13}N_{3}+\text{NaBr}$$

The theoretical AE was computed as follows

$$\mathrm{AE}\;\left(\%\right)=\frac{{\mathrm{MW}}_{\text{product}}}{\sum {\mathrm{MW}}_{\text{reactants}}}\times 100=69.6\%$$

The experimental efficiency was evaluated in terms of the reaction mass efficiency (RME), specific process mass intensity (sPMI), and E‐factor, using the actual stoichiometric ratios (1.15 equiv of benzyl bromide, 1.50 equiv of sodium azide, and 1.00 equiv of phenylacetylene). Solvents and catalysts were excluded from the calculations.

Definitions

RME=(mass of product/total mass of reactants)×100

sPMI=(total mass of reactants)/(mass of product)

$$\begin{array}{cc} E\text{‐}\mathrm{factor} & =(\text{mass of waste})/(\text{mass of product})\\ & =(\mathrm{reactants}-\mathrm{product})/\mathrm{product} \end{array}$$

Table 5 highlights the tradeoffs between yield, solvent choice, and mass‐based metrics. The DMF/water benchmark (entry 1) provided an 82% yield with an RME of 48.68%; however, DMF is a less‐preferred dipolar aprotic solvent, and its replacement with more sustainable alternatives is strongly encouraged [35]. Switching to water as the sole reaction medium (entry 2) maintained a high yield (86%), while slightly improving the overall mass‐based performance, with an RME of 51.05%, an sPMI of 1.96, and an E‐factor of 0.96. Under ultrasound with minimal water (*η* = 2), the protocol preserved competitive metrics while reducing reaction time (entry 3). Using the Carrara‐derived catalyst under the same sonication conditions (entry 4) gave a moderate yield (70%) with still acceptable mass intensities, illustrating a viable route to upcycle quarry residues into functional catalytic materials.

## 3 Conclusions

In conclusion, we demonstrated that a deliberately simple room‐temperature adsorption step is sufficient to transform benign carbonate minerals into practical and recyclable copper catalysts for click chemistry. The resulting CaCO_3_–Cu and Carrara–Cu materials promoted fast and selective CuAAC in neat water under mild sonochemical activation, using glucose as a benign reductant to generate the active Cu(I) species in situ*.* Under these conditions, benzyl bromides/azide/alkynes were converted to 1,4‐disubstituted 1,2,3‐triazoles in short reaction times (typically 1 h at 30 °C). Simultaneously, catalyst handling remained straightforward: the solid could be recovered by simple filtration and reused, allowing copper to be intercepted, retained, and deployed repeatedly rather than being lost to the solution. Beyond its performance, the Carrara marble powder case conveys a particularly resonant sustainability message that merits exploration. Carrara marble powders, a stone‐industry waste, can be repurposed as a value‐added support for recyclable CuAAC catalysis. Here, these residues are repurposed as functional catalyst supports, turning an industrial byproduct into a reusable platform for modern synthesis. Compared with previously reported heterogeneous CuAAC catalytic systems, the present methodology combines several advantageous features, including the use of water as the sole reaction medium, mild operating conditions (30 °C), straightforward catalyst preparation through simple Cu(II) adsorption onto carbonate supports, and catalyst recovery by filtration. While some heterogeneous CuAAC protocols reported in the literature achieve higher yields, they frequently rely on more complex catalyst syntheses, elevated temperatures, organic solvents, or engineered support materials [17, 18, 19]. In contrast, the CaCO_3_–Cu system developed herein employs abundant and inexpensive carbonate sources, including Carrara marble waste, providing a practical and sustainable alternative for triazole synthesis within a circular‐economy framework. Taken together, this study links waste valorization (stone‐industry residues), benign reaction media (water) and low‐energy activation (ultrasound at 30 °C) into an operationally simple click methodology that avoids hazardous solvents, reduces energy demand, and advances the practical case for recyclable heterogeneous CuAAC.

## Author Contributions

**Riccardo Serra:** writing – original draft, methodology, investigation, formal analysis, software. **Dipak J. Fartade:** writing – review and editing, methodology, investigation, data curation, software. **Pietro Caboni:** methodology, investigation, formal analysis. **Cristina Carucci:** writing – original draft, writing – review and editing, methodology, investigation, formal analysis, data curation. **Andrea Salis:** writing – review and editing, supervision, resources. **Enrica Tuveri**: investigation, methodology. **Antonella Rossi:** writing – review and editing, supervision, resources. **Andrea Citarella:** writing – review and editing, methodology, investigation, formal analysis, conceptualization, data curation. **Andrea Porcheddu:** writing – review and editing, methodology, investigation, formal analysis, supervision, resources, project administration, funding acquisition, conceptualization, visualization.

## Funding

This work was supported by Ministero dell’Ambiente e della Sicurezza Energetica (Non serviti Call (2021 edition), Line 3, CUP F33C23000680001) and the Italian Ministry of University and Research (MUR) through the National Recovery and Resilience Plan (NRRP), funded by the European Union – Next Generation EU, under the project ECS0000038 – eINS Ecosystem of Innovation for Next Generation Sardinia (CUP F53C22000430001; Grant Assignment Decree No. 1056).

## Conflict of Interest

The authors declare no conflicts of interest.

## Supporting information

Supporting information to this article can be found online, including experimental procedures, copies of ^1^H NMR and ^13^C NMR spectra of the compounds, and Figure S1.

## Data Availability

Data will be made available on request.
