# Supplementary material for: Copper on Calcium Carbonate and Marble Waste as Recyclable Catalysts for Ultrasound‐Assisted One‐Pot Synthesis of 1,4‐Disubstituted 1,2,3‐Triazoles
Source: ChemSusChem. 2026 Aug 19;19(16):e70944. doi: 10.1002/cssc.70944 (PMC13489785; doi:10.1002/cssc.70944)
Supplement: Supplementary file 1 [file CSSC-19-e70944-s001.pdf]

Supplementary materials for:

Copper on $CaCO_3$ and Marble Waste as Recyclable Catalysts for Ultrasound-Assisted One-Pot Synthesis of 1,4-Disubstituted 1,2,3–Triazoles

Riccardo Serra,[a] Dipak J. Fartade,[a] Pietro Caboni,[a] Cristina Carucci,[a] Andrea Salis,[a] Enrica Tuveri,[a] Antonella Rossi, [a] Andrea Citarella,[a,*] Andrea Porcheddu[a,*]

Department of Chemical and Geological Sciences, University of Cagliari, Cittadella Universitaria di Monserrato, SS 554, bivio per Sestu, 09042 Monserrato (CA), Italy

* Corresponding authors

E-mail addresses: andrea.citarella@unica.it (A. Citarella); porcheddu@unica.it (A. Porcheddu)

Contents	Pages
**Materials and methods**	1
**Catalyst preparation**	1
**Physico-chemical characterization**	2
**General procedure for the synthesis of 1,2,3–triazoles**	2
**Spectral and Characterization Data**	3 – 5
**Catalyst Recycling Procedure**	6
**^{1}H and ^{13}C NMR Spectra**	7 – 19
**Figure S1**	20
**Figure S2**	21
**References**	22

1. Materials and Methods

A commercial calcium carbonate ($CaCO_3$) powder with a particle size of approximately 14 μm and a fine Carrara marble powder ($CaCO_3$) with a particle size in the range of 38–45 μm were used in this study. The difference in particle size distribution between the two powders was taken into account in the interpretation of the results. Prior to use, both powders were washed with analytical-grade ethyl alcohol and subsequently dried in an oven at 110 °C for 24 h. Commercially available reagents were purchased from Acros, Aldrich, Strem Chemicals, Alfa-Aesar, TCI Europe and used as received. Reactions were carried out in an ultrasonic bath (VWR Ultrasonic Cleaner, model USC 300 TH, 45 kHz, 80 W) equipped with temperature control; the bath temperature was set to 30 °C. All reactions were monitored by thin-layer chromatography (TLC) performed on glass-backed silica gel 60 F_{254}, 0.2 mm plates (Merck), and compounds were visualized under UV light (254 nm) or using cerium ammonium molybdate solution with subsequent heating. The eluents were technical grade. ^{1}H and ^{13}C liquid NMR spectra were recorded on a Bruker Avance III HD 600 MHz NMR spectrometer at 298 K and were calibrated using trimethylsilane (TMS). Proton chemical shifts are expressed in parts per million (ppm, δ scale) and are referred to as the residual hydrogen in the solvent ($CHCl_3$, 7.27 ppm or DMSO-d_6 2.54 ppm). Data are represented as follows: chemical shift, multiplicity (s = singlet, d = doublet, t = triplet, q = quartet, m = multiplet and/or multiple resonances, br s = broad singlet, and combination of thereof), coupling constant (J) in Hertz (Hz) and integration. Carbon chemical shifts are expressed in parts per million (ppm, δ scale) and are referenced to the carbon resonances of the NMR solvent ($CDCl_3$, δ 77.0 ppm or δ DMSO-d_6 δ 39.5 ppm). Deuterated NMR solvents were obtained from Aldrich. High resolution mass spectra (HRMS) were recorded on LTQ Orbitrap Elite (Thermo Fischer Scientific) instrument (ESI). FT-IR analyses were registered with Perkin Elmer Spectrum 100 FT-IR Spectrometer (Attenuated Total Reflectance Infrared ATR-IR device) in reflectance mode. Measurements were performed directly on the powders. Yields refer to pure isolated materials when feasible. Otherwise, only the conversion ratio has been reported.

2. Catalyst preparation

$CaCO_3$ (200 mg) was combined with a 0.02 M $CuSO_4$ solution, and the mixture was stirred for 24 hours at room temperature. Following this period, UV-Visible spectra were recorded to assess the concentration of copper remaining in the solution. The UV-Visible mass balance indicated that the supported catalyst had a copper loading of approximately 1.32 mmol g^{-1}** (≈ 0.132 mmol per 100 mg, ~8.4 wt% Cu). Measurements were conducted using a Thermo Nicolet Evolution 300 double-beam spectrophotometer, equipped with 1 cm quartz cuvettes, operating in the 190–1100 nm range at ambient temperature. Each sample spectrum was recorded against a blank reference after baseline

correction using the same solvents. The reduction or disappearance of the characteristic Cu(II) absorption bands confirmed the effective adsorption of copper onto the carbonate surface, leading to the formation of the supported catalyst ($CaCO_3$–Cu). The UV-Visible mass balance showed that the supported catalyst had a copper loading of approximately 1.32 mmol g^{-1} (≈ 0.132 mmol per 100 mg, ~8.4 wt% Cu), derived from the reduction in $CuSO_4$ concentration from 20 mM to 6.8 mM in 20 mL when in contact with 200 mg $CaCO_3$.

3. Physico-chemical characterization

$CaCO_3$ samples before and after Cu^{2+} adsorption and after click chemistry were characterized through scanning electron microscopy, energy dispersive X-ray spectroscopy (SEM-EDX), thermogravimetric analysis (TGA) and electrophoretic light scattering (ELS, zeta potential). Zeta potential (ζ) has been measured by electrophoretic light scattering (ELS) by mean of Zetasizer Nano ZSP (Malvern Instruments) with phase analysis light scattering (PALS) in a back scatter configuration geometry, at laser wavelength of λ= 633 nm. The samples were prepared in water at 0.1 mg mL^{-1} and sonicated for 5 minutes. pH was measured after dispersion and was not adjusted or buffered. Mass loss values were extracted from baseline corrected TGA curves (mass loss %) defining the 3 degradation regions. TGA measurements were carried out in the T range 25–850 °C (heating rate = 10 °C/min), and under oxygen flow by means of a Perkin Elmer TGA/DSC.

4. General procedure for the synthesis of 1,2,3–triazoles

In a glass vial, the appropriate benzyl bromide (1.15 mmol), alkyne (1.0 mmol), sodium azide (1.5 mmol), glucose (0.2 mmol, 20 mol%), and $CaCO_3$–Cu catalyst (40 mg) were combined with distilled water ($\eta = 2$, typically 0.8 – 1.0 mL). The vial was placed in an ultrasonic bath and sonicated at 30 °C for 1 h. The progress of the reaction was monitored using TLC. After the reaction was completed, the mixture was diluted with ethyl acetate, and the catalyst was recovered by filtration, washed with ethyl acetate and dried under *vacuum* for reuse. The combined organic phases were washed with brine, dried over anhydrous sodium sulfate (Na_2SO_4) and concentrated under reduced pressure to afford the corresponding 1,4-disubstituted-1,2,3-triazole. All products are known compounds; their melting points and ^{1}H NMR spectra were consistent with the literature data.

5. Spectral and Characterization Data

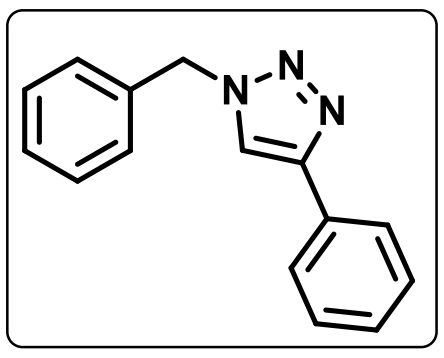

1-Benzyl-4-phenyl-1*H*-1,2,3-triazole (1). White powder; m.p. = 124–125 °C. 81% yield. ^{1}H NMR (600 MHz, $CDCl_3$): δ 7.81 (d, *J* = 7.6 Hz, 2H), 7.71 (s, 1H), 7.42 – 7.33 (m, 5H), 7.33 – 7.28 (m, 3H), 5.56 (s, 2H). ^{13}C NMR (150 MHz, $CDCl_3$): δ 148.4, 134.8, 130.7, 129.2, 128.9, 128.8, 128.3, 128.1, 125.8, 119.8, 54.4. HRMS (ESI), *m/z* $[M+H]^+$: calculated for $C_{15}H_{14}N_3^+$ 236.1182; found 236.1191. The compound characterization is in agreement with that previously reported.[1]

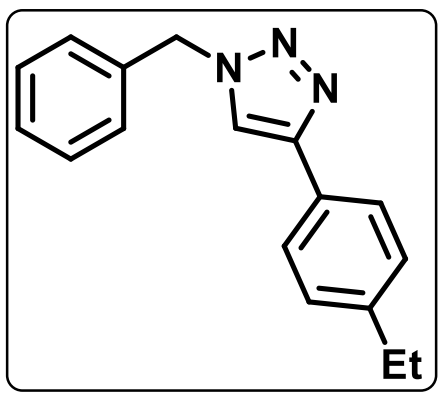

1-Benzyl-4-(4-ethylphenyl)-1*H*-1,2,3-triazole (2). White powder; m.p. = 117–121 °C (lit. 121–123 °C). 77% yield. ^{1}H NMR (600 MHz, $CDCl_3$): δ 7.75 (d, *J* = 7.8 Hz, 2H), 7.67 (s, 1H), 7.50 – 7.21 (m, 7H), 5.57 (s, 2H), 2.69 (q, *J* = 8.0 Hz, 2H), 1.28 (t, *J* = 7.8 Hz, 3H). ^{13}C NMR (150 MHz, $CDCl_3$): δ 148.4, 144.4, 134.8, 129.2, 128.8, 128.4, 128.0, 125.8, 119.3, 54.2, 28.7, 15.6. HRMS (ESI), *m/z* $[M+H]^+$: calculated for $C_{17}H_{18}N_3^+$ 264.1495; found 264.1410. The compound characterization is in agreement with that previously reported.[2]

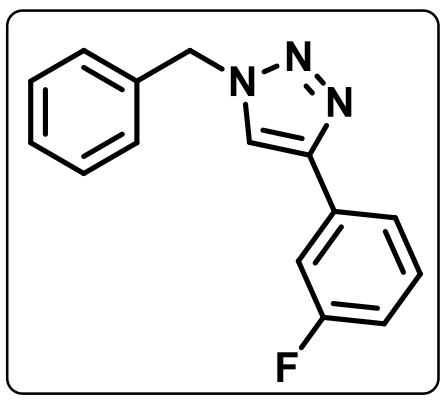

1-Benzyl-4-(3-fluorophenyl)-1*H*-1,2,3-triazole (3). Brown powder; m.p. = 107–111 °C (lit. 109–111 °C). 71% yield. ^{1}H NMR (600 MHz, $CDCl_3$): δ 7.76 (s, 1H), 7.56 (dd, *J* = 8.2 Hz, 2H), 7.42 – 7.29 (m, 6H), 7.01 (t, *J* = 8.2 Hz, 1H), 5.58 (s, 2H). ^{13}C NMR (150 MHz, $CDCl_3$): δ 163.3 (d, $J_{C,F}$ = 254.6 Hz), 134.6, 133.0 (d, $J_{C,F}$ = 8.2 Hz), 130.50 (d, $J_{C,F}$ = 8.2 Hz), 129.3, 129.0, 128.3, 121.4, 115.1 (d, $J_{C,F}$ = 21.5 Hz), 112.7 (d, $J_{C,F}$ = 21.5 Hz), 54.6. HRMS (ESI), *m/z* $[M+H]^+$: calculated for $C_{15}H_{13}FN_3^+$ 254.1088; found 254.1117. The compound characterization is in agreement with that previously reported.[3]

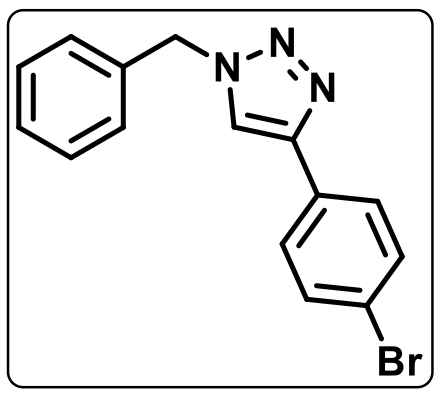

1-Benzyl-4-(4-bromophenyl)-1*H*-1,2,3-triazole (4). Brown powder; m.p. = 148–150 °C (lit 149 °C). 51% yield. ^{1}H NMR (600 MHz, $CDCl_3$): δ 7.66 (d, *J* = 7.8 Hz, 2H), 7.51 (d, *J* = 7.8 Hz, 2H), 7.38 (q, *J* = 7.0, 6.6 Hz, 3H), 7.30 (d, *J* = 6.9 Hz, 2H), 5.56 (s, 2H). ^{13}C NMR (150 MHz, $CDCl_3$): δ 147.3, 134.6, 132.0, 129.6, 129.3, 129.0, 128.2, 127.3, 122.1, 119.7, 54.4. HRMS (ESI), *m/z* $[M+H]^+$: calculated for $C_{15}H_{13}BrN_3^+$ 314.0287; found 314.0203. The compound characterization is in agreement with that previously reported.[4]

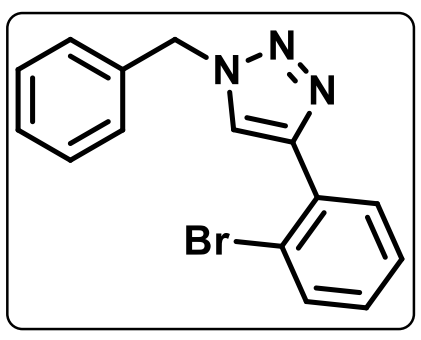

1-Benzyl-4-(2-bromophenyl)-1*H*-1,2,3-triazole (5). Brown amorphous solid. 60% yield. ^{1}H NMR (600 MHz, $CDCl_3$): δ 8.06 (s, 1H), 8.01 (d, *J* = 7.8, 1H), 7.54 – 7.49 (m, 1H), 7.27 (m, 4H), 7.21 (d, *J* = 7.8 Hz, 2H), 7.11 – 7.04 (m, 1H), 5.50 (s, 2H). ^{13}C NMR (150 MHz, $CDCl_3$): δ 145.8, 134.7, 133.5, 131.3, 130.6, 129.4,

129.2, 128.8, 128.0, 127.7, 123.1, 121.2, 54.3. HRMS (ESI), *m/z* $[M+H]^+$: calculated for $C_{15}H_{13}BrN_3^+$ 314.0287; found 314.0306. The compound characterization is in agreement with that previously reported.[5]

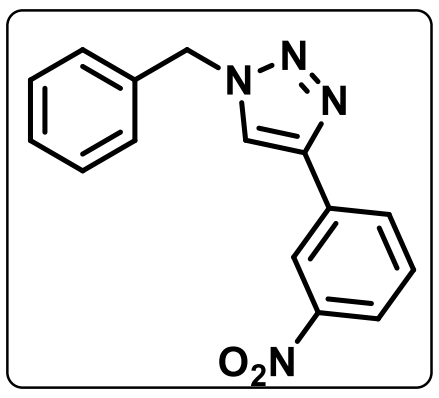

1-Benzyl-4-(3-nitrophenyl)-1*H*-1,2,3-triazole (6). Brown powder; m.p. = 136–140 °C (lit. 134–136 °C). 68% yield. ^1H NMR (600 MHz, $CDCl_3$): δ 8.56 (d, *J* = 2.3 Hz, 1H), 8.24 (d, *J* = 8.2 Hz, 1H), 8.16 (dd, *J* = 8.2, 1.8 Hz, 1H), 7.83 (s, 1H), 7.59 (t, *J* = 8.2 Hz, 1H), 7.41 (m, 4H), 7.37 – 7.31 (m, 2H), 5.61 (s, 2H). ^{13}C NMR (150 MHz, $CDCl_3$): δ 148.8, 134.3, 132.5, 131.6, 130.0, 129.5, 129.2, 129.1, 128.4, 128.1, 122.9, 120.6, 54.7. HRMS (ESI), *m/z* $[M+H]^+$: calculated for $C_{15}H_{13}N_4O_2^+$ 281.1033; found 281.1074. The compound characterization is in agreement with that previously reported.[6]

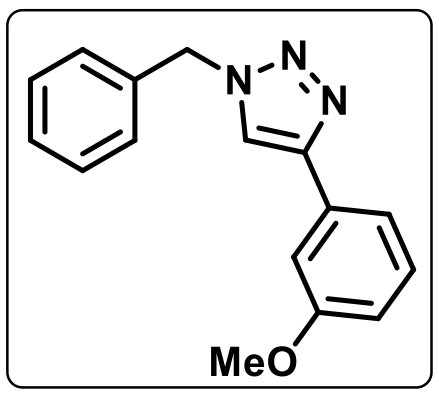

1-Benzyl-4-(3-methoxyphenyl)-1*H*-1,2,3-triazole (7). Brown powder; m.p. = 80–82 °C (lit. 79–81 °C). 83% yield. ^1H NMR (600 MHz, $CDCl_3$): δ 7.75 (s, 1H), 7.44 (d, *J* = 11.7 Hz, 1H), 7.40 – 7.24 (m, 7H), 6.86 (d, *J* = 8.1 Hz, 1H), 5.55 (s, 2H), 3.84 (s, 3H). ^{13}C NMR (150 MHz, $CDCl_3$): δ 160.0, 148.3, 134.7, 131.9, 129.8, 129.1, 128.7, 128.1, 118.5, 118.1, 114.3, 110.7, 55.4, 54.3. HRMS (ESI), *m/z* $[M+H]^+$: calculated for $C_{16}H_{16}N_3O^+$ 266.1288; found 266.1197. The compound characterization is in agreement with that previously reported.[3]

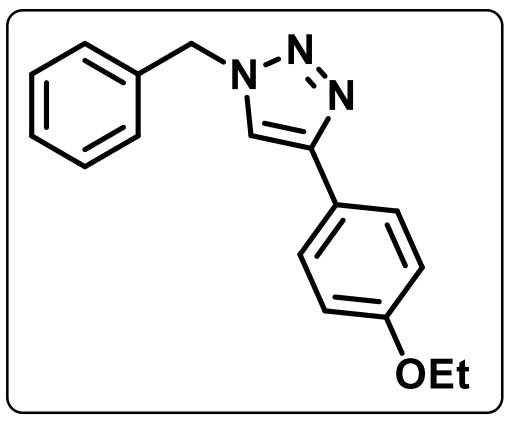

1-Benzyl-4-(4-ethoxyphenyl)-1*H*-1,2,3-triazole (8). Withish powder; m.p. = 146–148 °C. 57% yield. ^1H NMR (600 MHz, $CDCl_3$): δ 7.94 – 7.68 (m, 2H), 7.37 (dq, *J* = 14.1, 7.3 Hz, 4H), 7.29 (d, *J* = 7.3 Hz, 2H), 6.94 (s, 2H), 5.56 (s, 2H), 4.06 (q, *J* = 7.0 Hz, 2H), 1.42 (t, *J* = 7.0 Hz, 3H). ^{13}C NMR (150 MHz, $CDCl_3$): δ 159.2, 148.2, 134.8, 129.3, 129.0, 128.3, 126.9, 123.3, 115.0, 77.4, 63.7, 54.9, 14.9. HRMS (ESI), *m/z* $[M+H]^+$: calculated for $C_{17}H_{18}N_3O^+$ 280.1444; found 280.1387. The compound characterization is in agreement with that previously reported.[7]

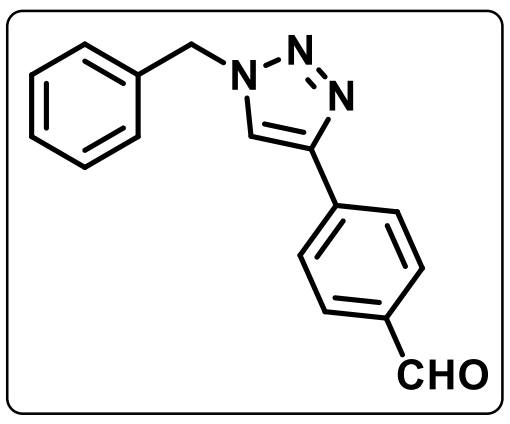

4-(1-Benzyl-1*H*-1,2,3-triazol-4-yl)benzaldehyde (10). Yellow powder; m.p. = 137–139 °C (lit. 138–140 °C). 78% yield. ^1H NMR (600 MHz, DMSO-d_6): δ 10.01 (s, 1H), 8.84 (s, 1H), 8.09 (d, *J* = 8.2 Hz, 2H), 7.98 (d, *J* = 8.2 Hz, 2H), 7.45 – 7.28 (m, 5H), 5.68 (s, 2H). 13C NMR (150 MHz, DMSO-d_6): δ 192.1, 145.2, 135.9, 135.4, 134.9, 129.9, 128.4, 127.9, 127.6, 125.2, 122.7, 52.8. HRMS (ESI), *m/z* $[M+H]^+$: calculated for $C_{16}H_{14}N_3O^+$ 264.1131; found 264.1189. The compound characterization is in agreement with that previously reported.[8]

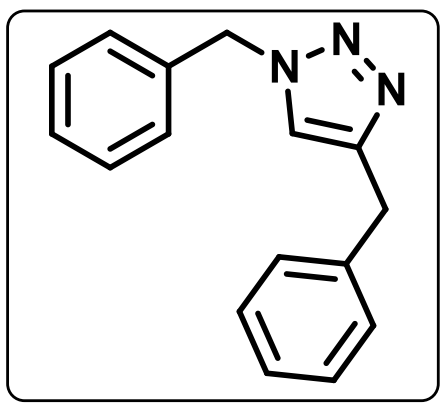

1,4-Dibenzyl-1*H*-1,2,3-triazole (11). White amorphous solid. 72% yield. ^{1}H NMR (600 MHz, $CDCl_3$): δ 7.40 – 7.31 (m, 3H), 7.32 – 7.16 (m, 8H), 5.48 (s, 2H), 4.07 (s, 2H). ^{13}C NMR (150 MHz, $CDCl_3$): δ 148.5, 138.9, 134.7, 129.2, 128.9, 128.8, 128.8, 128.1, 126.7, 54.5, 32.3. The compound characterization is in agreement with that previously reported.[9]

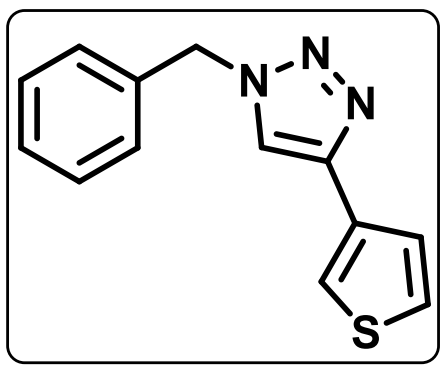

1-Benzyl-4-(thiophen-3-yl)-1*H*-1,2,3-triazole (12). Yellow amorphous solid. 71% yield. ^{1}H NMR (600 MHz, $CDCl_3$): δ 7.70 (s, 1H), 7.44 – 7.34 (m, 4H), 7.31 (d, J = 7.6 Hz, 2H), 7.28 (d, J = 5.0 Hz, 1H), 7.06 (d, J = 4.9 Hz, 1H), 5.56 (s, 2H). ^{13}C NMR (150 MHz, $CDCl_3$): δ 144.5, 134.6, 133.0, 129.3, 129.0, 128.2, 127.7, 125.2, 124.4, 119.7, 54.5. The compound characterization is in agreement with that previously reported.[10]

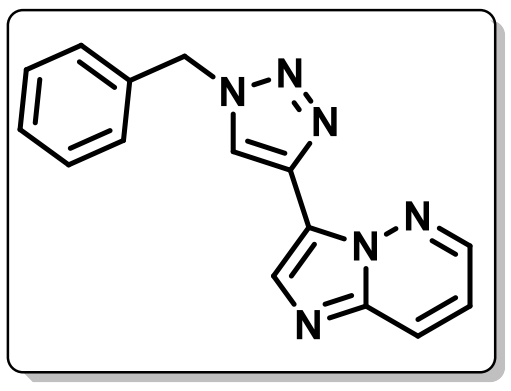

3-(1-Benzyl-1*H*-1,2,3-triazol-4-yl)imidazo[1,2-*b*]pyridazine (13). Brown amorphous solid. 67% yield. ^{1}H NMR (600 MHz, $CDCl_3$): δ 8.49 (s, 1H), 8.47 (dd, J = 4.5, 1.4 Hz, 1H), 8.39 (s, 1H), 8.18 (dd, J = 9.1, 1.4 Hz, 1H), 7.42 – 7.36 (m, 3H), 7.36 – 7.33 (m, 2H), 7.16 (dd, J = 9.1, 4.5 Hz, 1H), 5.66 (s, 2H). ^{13}C NMR (150 MHz, $CDCl_3$): δ 143.9, 139.2, 137.1, 134.8, 130.8, 129.3, 129.0, 128.1, 125.7, 121.7, 121.1, 117.3, 54.5. HRMS (ESI), *m/z* $[M+H]^+$: calculated for $C_{15}H_{13}N_6^+$ 277.1196; found 277.1204.

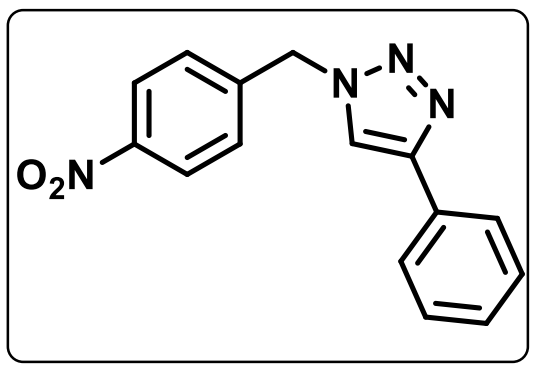

1-(4-Nitrobenzyl)-4-phenyl-1*H*-1,2,3-triazole (14). White solid; m.p. = 140 °C (lit. 139–142 °C). 68% yield. ^{1}H NMR (600 MHz, $CDCl_3$): δ 8.24 (d, J = 8.0 Hz, 2H), 7.81 (d, J = 8.0 Hz, 2H), 7.75 (s, 1H), 7.50 – 7.39 (m, 4H), 7.34 (t, J = 7.4 Hz, 1H), 5.70 (s, 2H). ^{13}C NMR (150 MHz, $CDCl_3$): δ 148.4, 147.8, 141.6, 129.9, 128.7, 128.3, 127.5, 125.5, 124.1, 119.6, 52.9. HRMS (ESI), *m/z* $[M+H]^+$: calculated for $C_{15}H_{13}N_4O_2^+$ 281.1033; found 281.1112. The compound characterization is in agreement with that previously reported.[1]

6. Catalyst Recycling Procedure

Upon completion of the reaction, the catalyst was recovered by filtration, washed with ethyl acetate and water (3 × 5 mL), dried under reduced pressure at room temperature, weighed and reused directly in the next catalytic run. No additional activation or copper replenishment was performed between cycles. Catalyst recovery was calculated as:

$$\text{Recovery (\%)} = (m_{recovered}/m_{initial}) \times 100$$

where $m_{recovered}$ is the mass of catalyst recovered after filtration and drying, and $m_{initial}$ is the mass of catalyst initially employed in the reaction (40 mg). Recycling experiments were performed under the optimized ultrasound-assisted conditions (Table 4, entry 5). The catalytic performance after each cycle was assessed by isolated product yield, while catalyst stability was evaluated by PXRD, FTIR, TGA and zeta potential measurements on selected recycled samples.

Table S1. Catalyst recovery during recycling experiments.

First Reuse		Second Reuse		Third Reuse		Fourth Reuse	
N°	Recovery	N°	Recovery	N°	Recovery	N°	Recovery
1	56.1%	1	56.1%	1	64.0%	1	73.1%
2	62.7%	2	39.7%	2	52.6%	2	62.0%
3	61.9%	3	52.4%	3	50.2%	3	56.4%
4	58.1%	4	64.9%	4	49.8%		
5	65.5%	5	60.0%	5	57.5%		
6	44.9%	6	61.1%	6	51.0%		
7	56.0%						
8	66.8%						
9	46.2%						
10	59.7%						
11	73.3%						
Yield ± SD	59% ± 8%	Yield ± SD	56% ± 9%	Yield ± SD	54% ± 6%	Yield ± SD	64% ± 8%

^{1}H and ^{13}C NMR spectra of the final compounds

^{1}H and ^{13}C NMR spectra of compound **1**

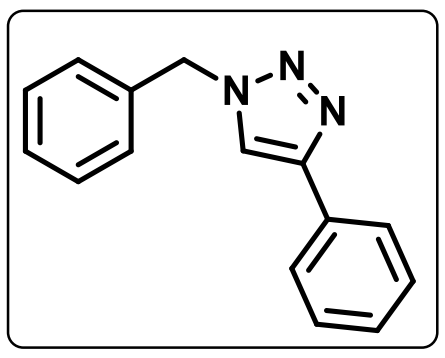

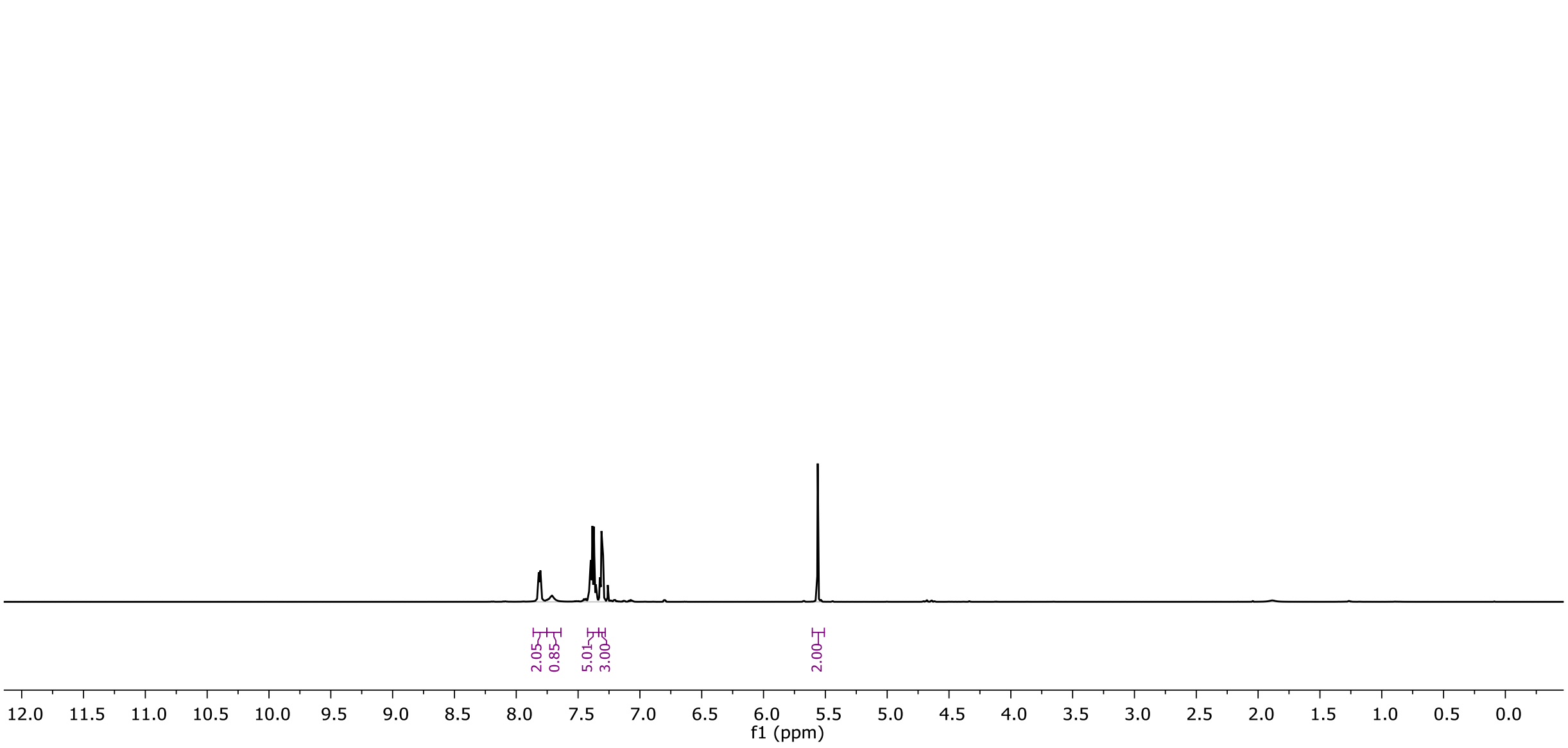

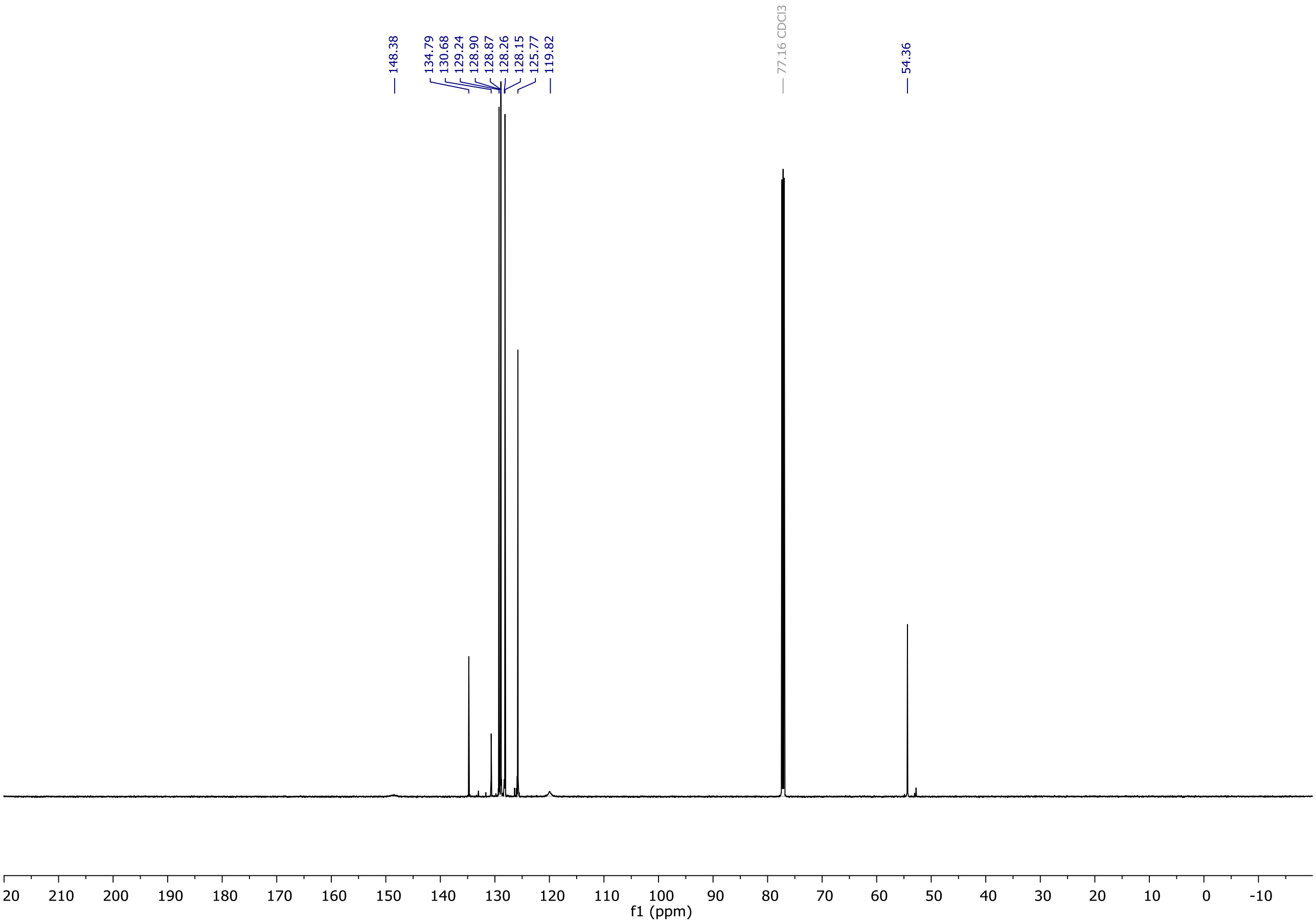

^{1}H and ^{13}C NMR spectra of compound **2**

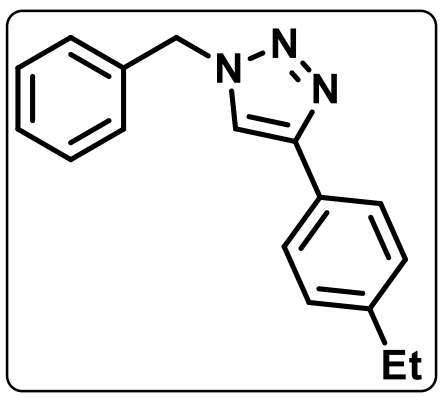

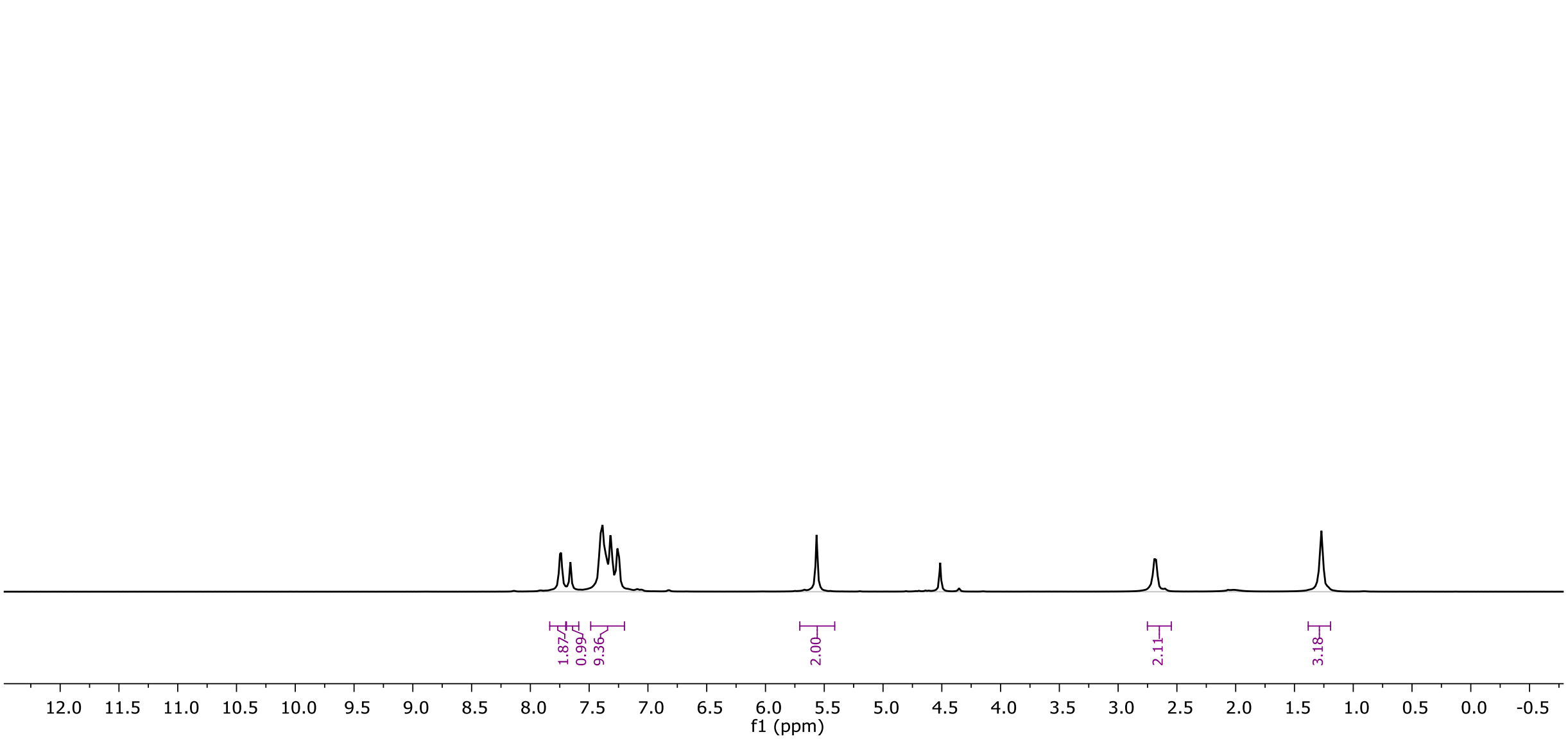

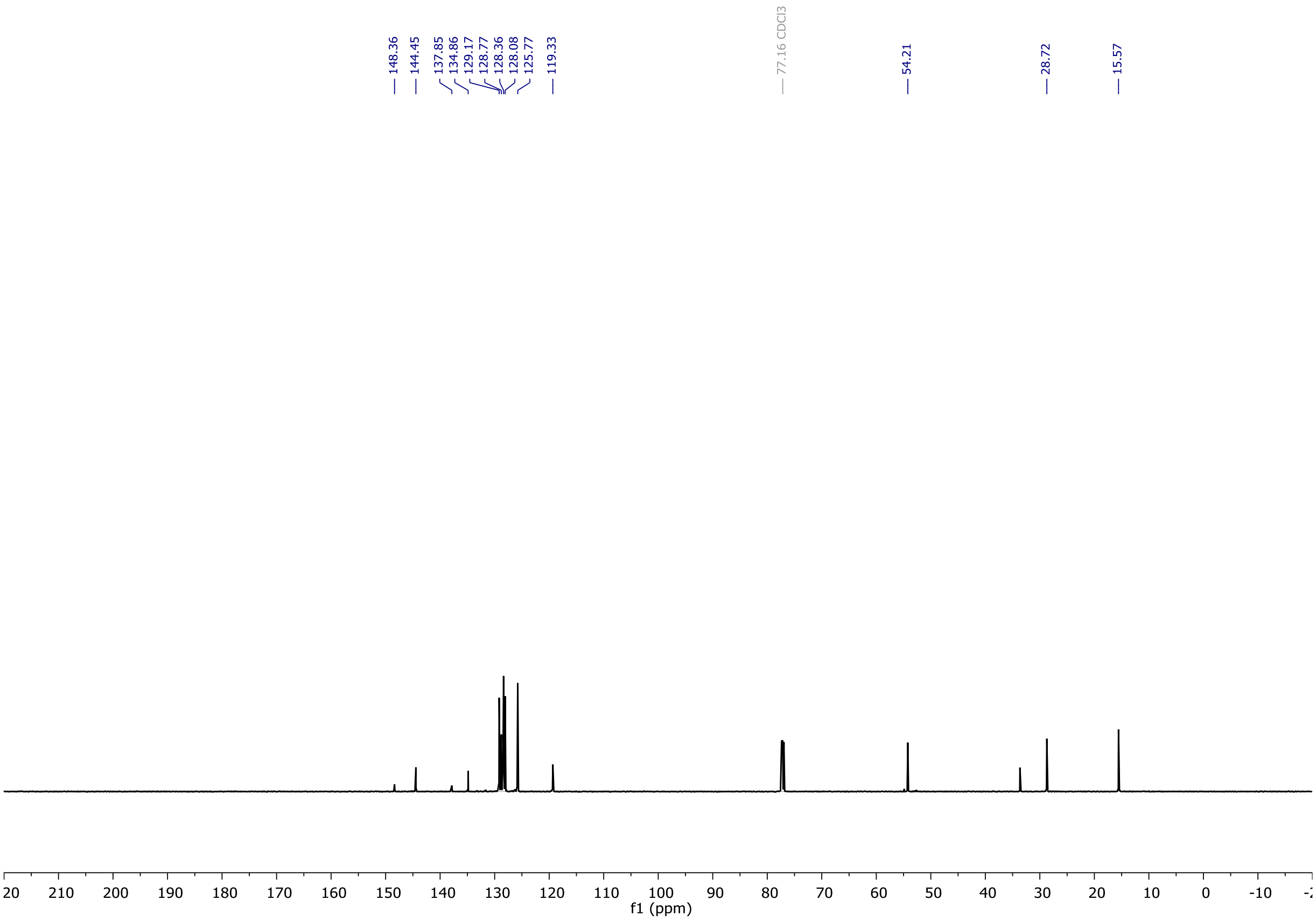

^{1}H and ^{13}C NMR spectra of compound **3**

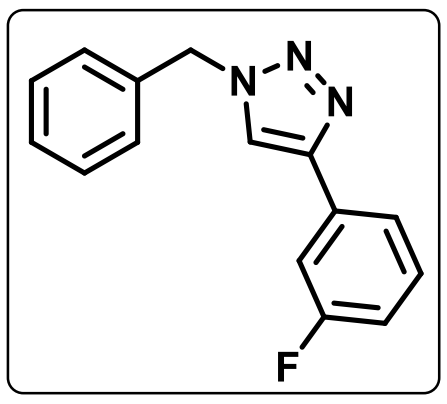

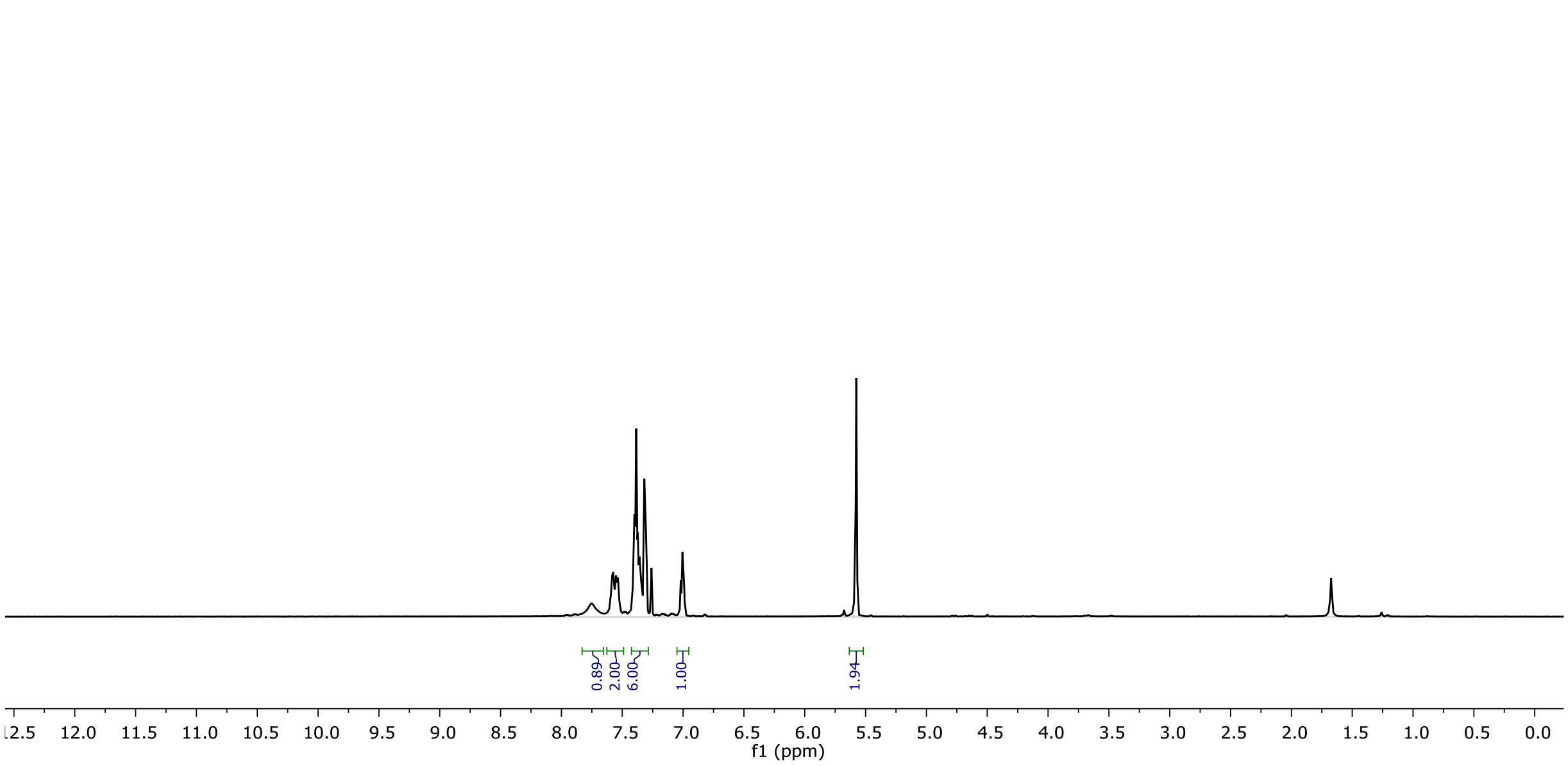

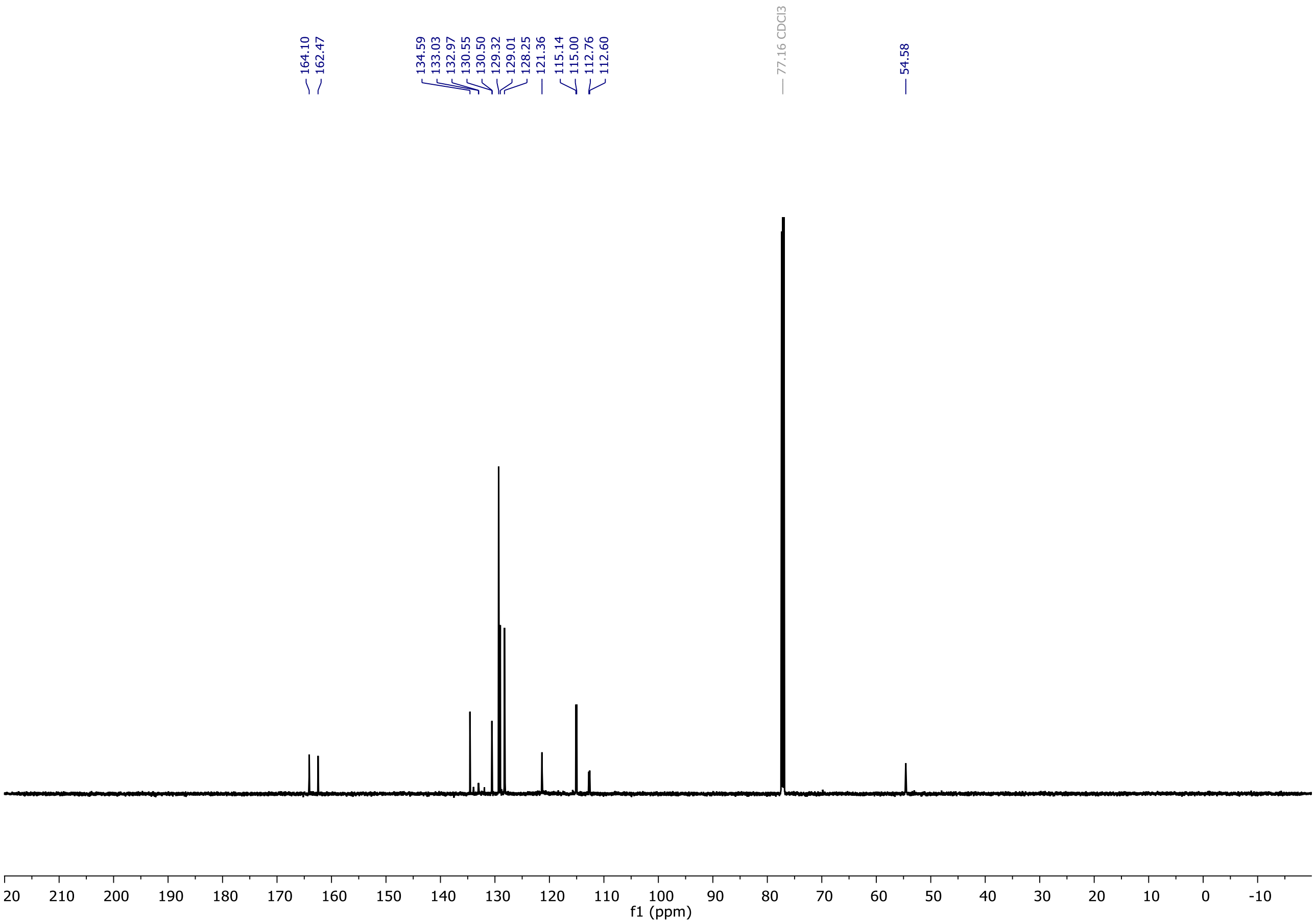

^{1}H and ^{13}C NMR spectrum of compound **4**

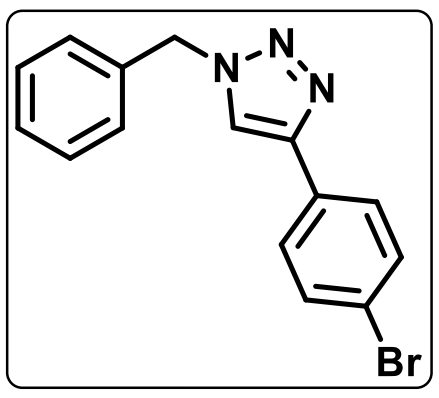

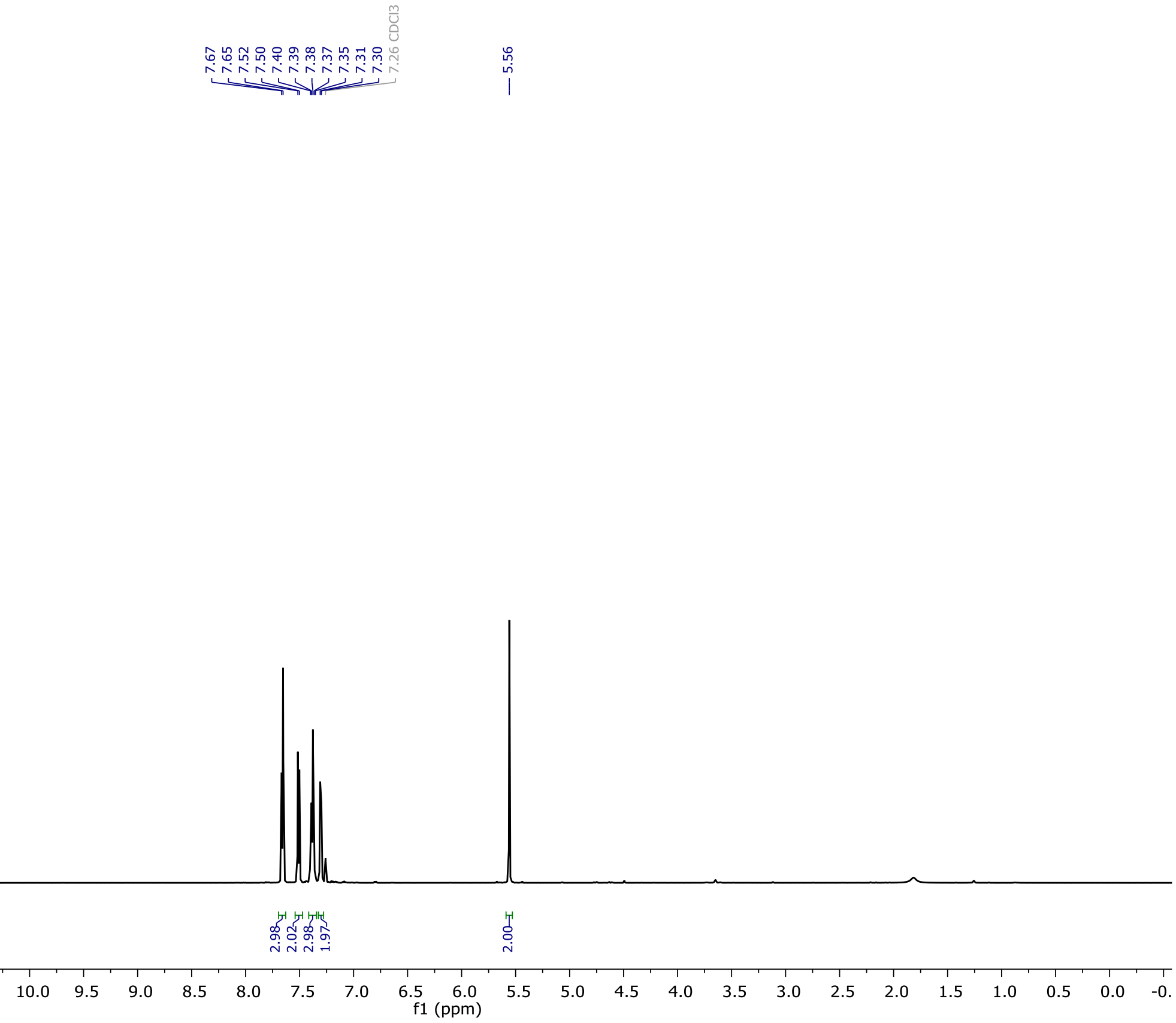

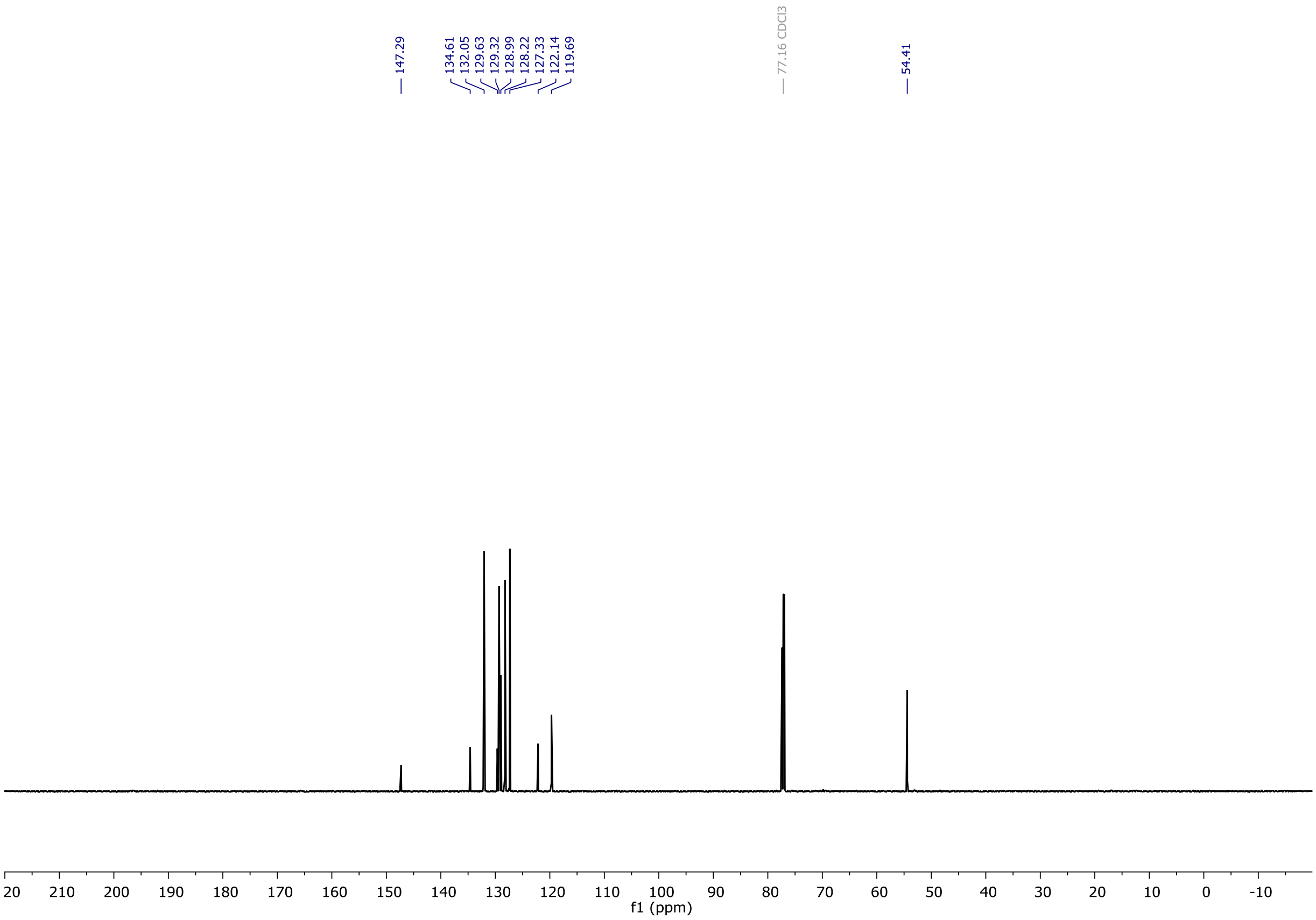

^{1}H NMR spectrum of compound **5**

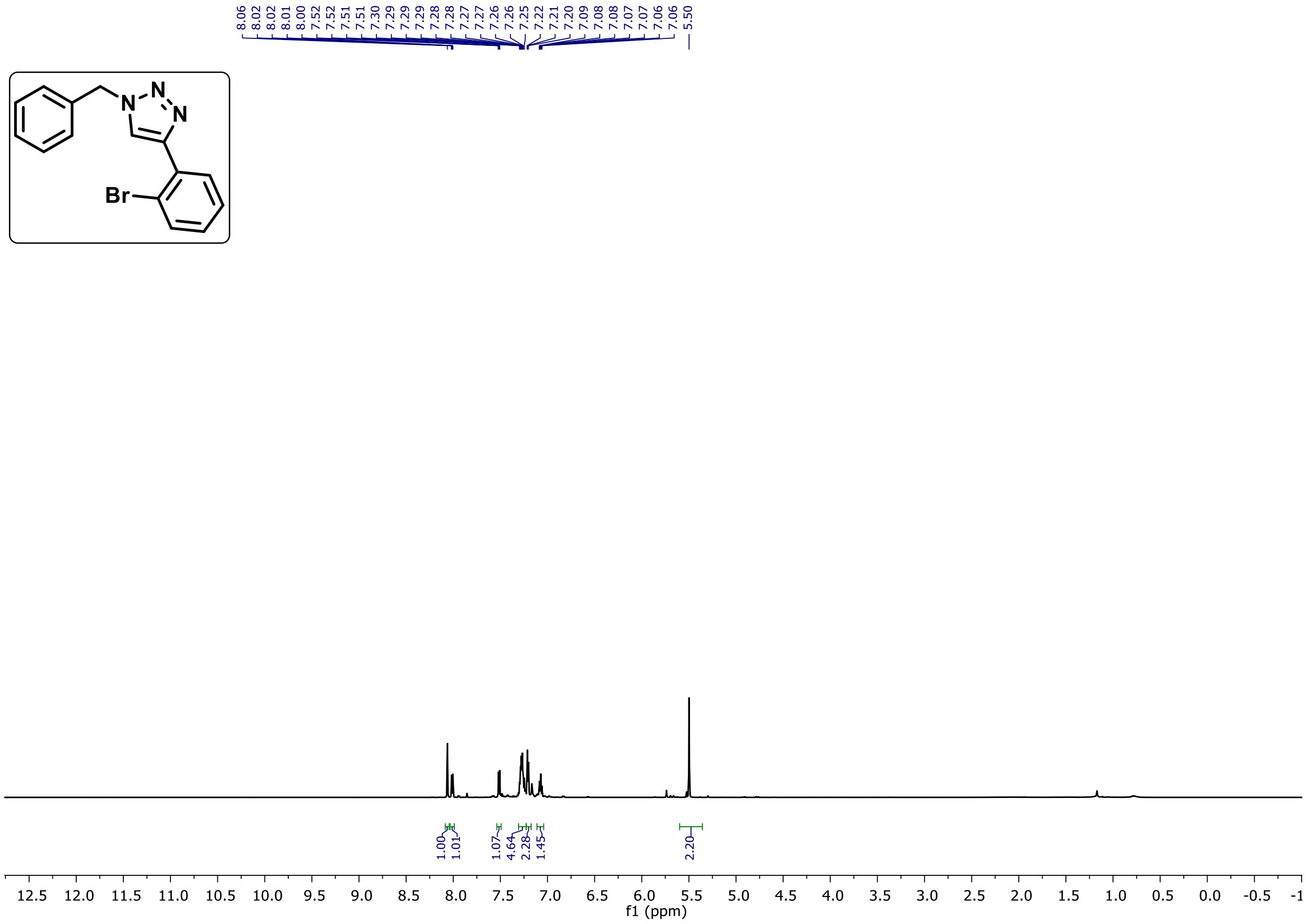

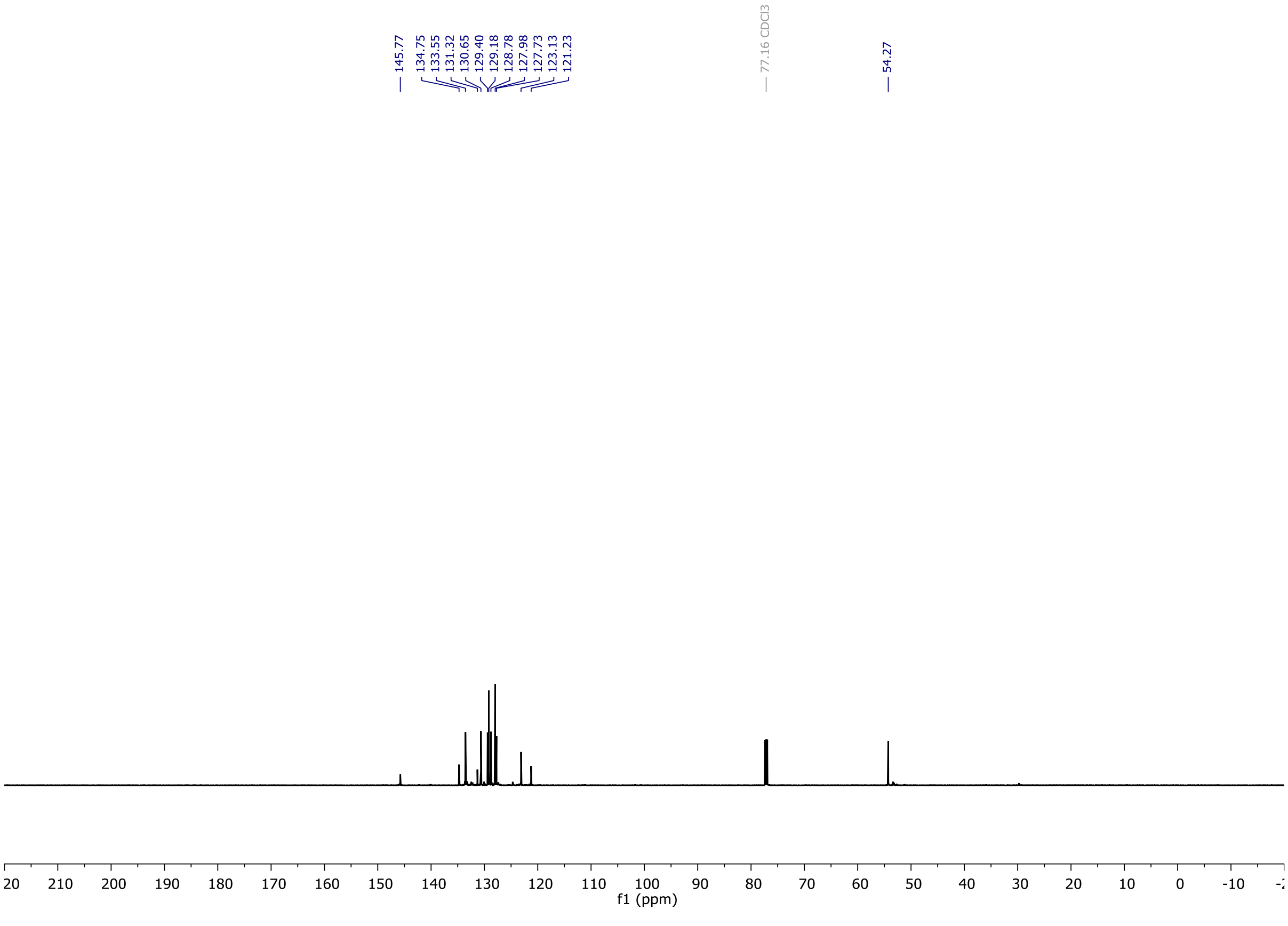

^{1}H and ^{13}C NMR spectra of compound **6**

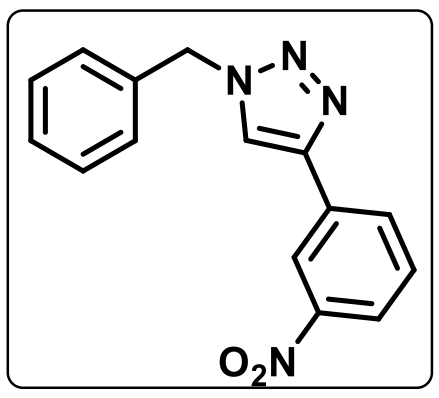

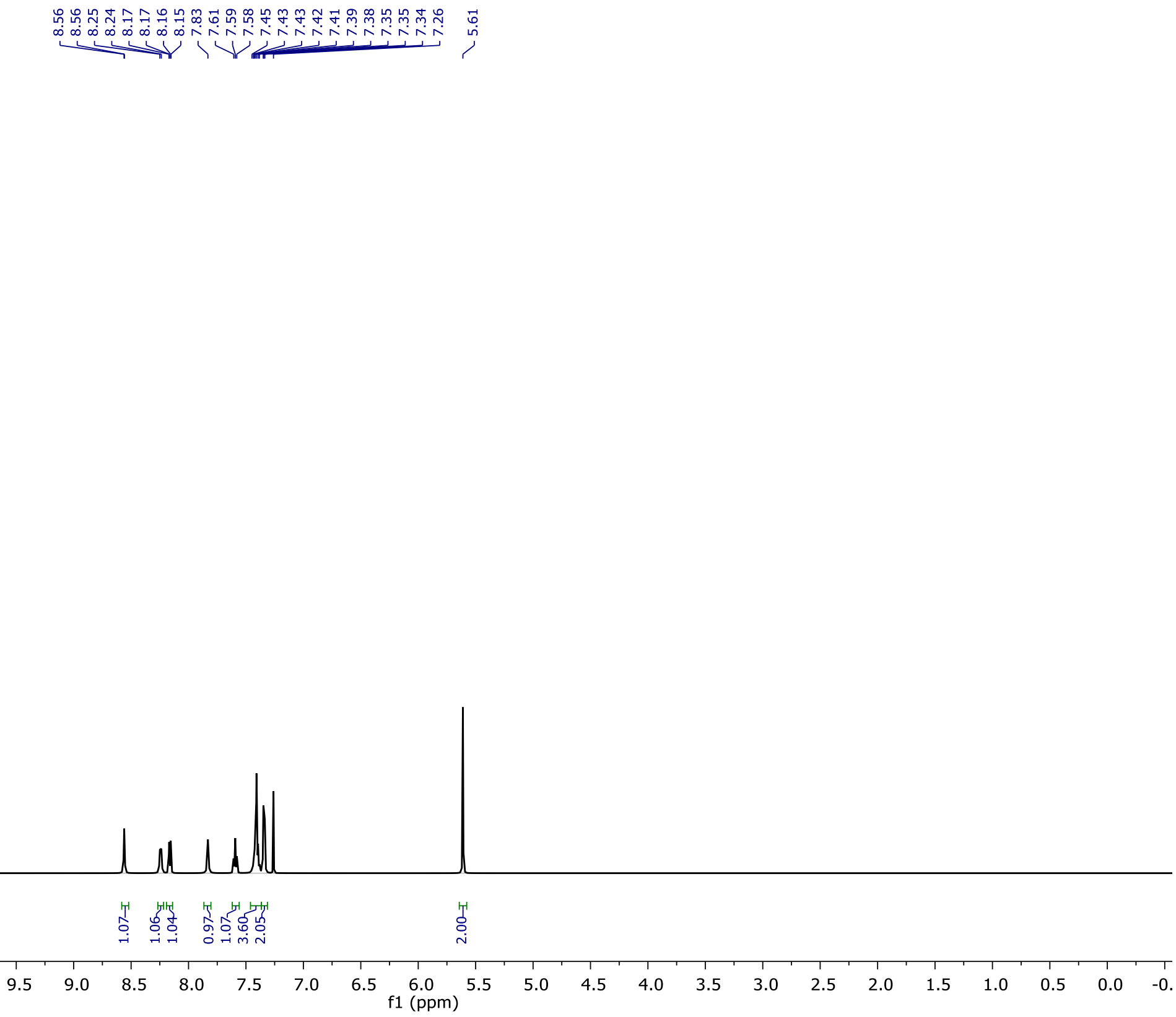

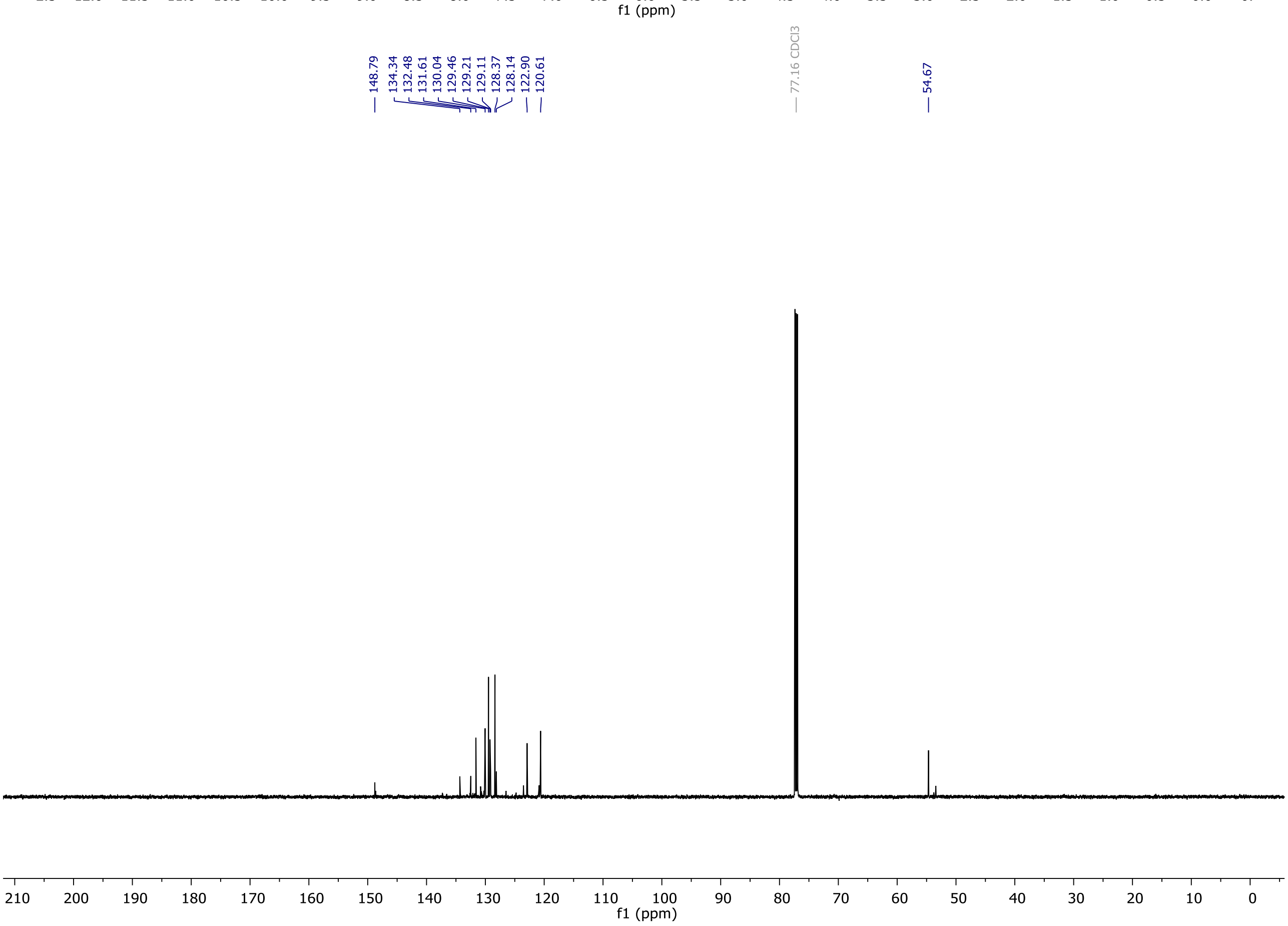

^{1}H and ^{13}C NMR spectra of compound **7**

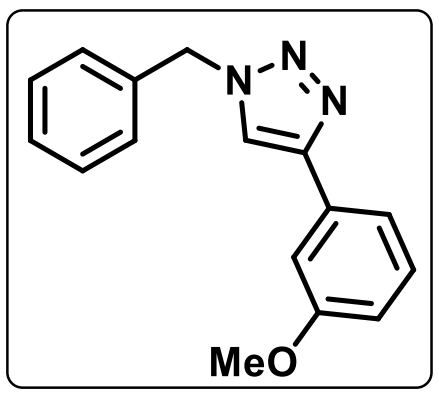

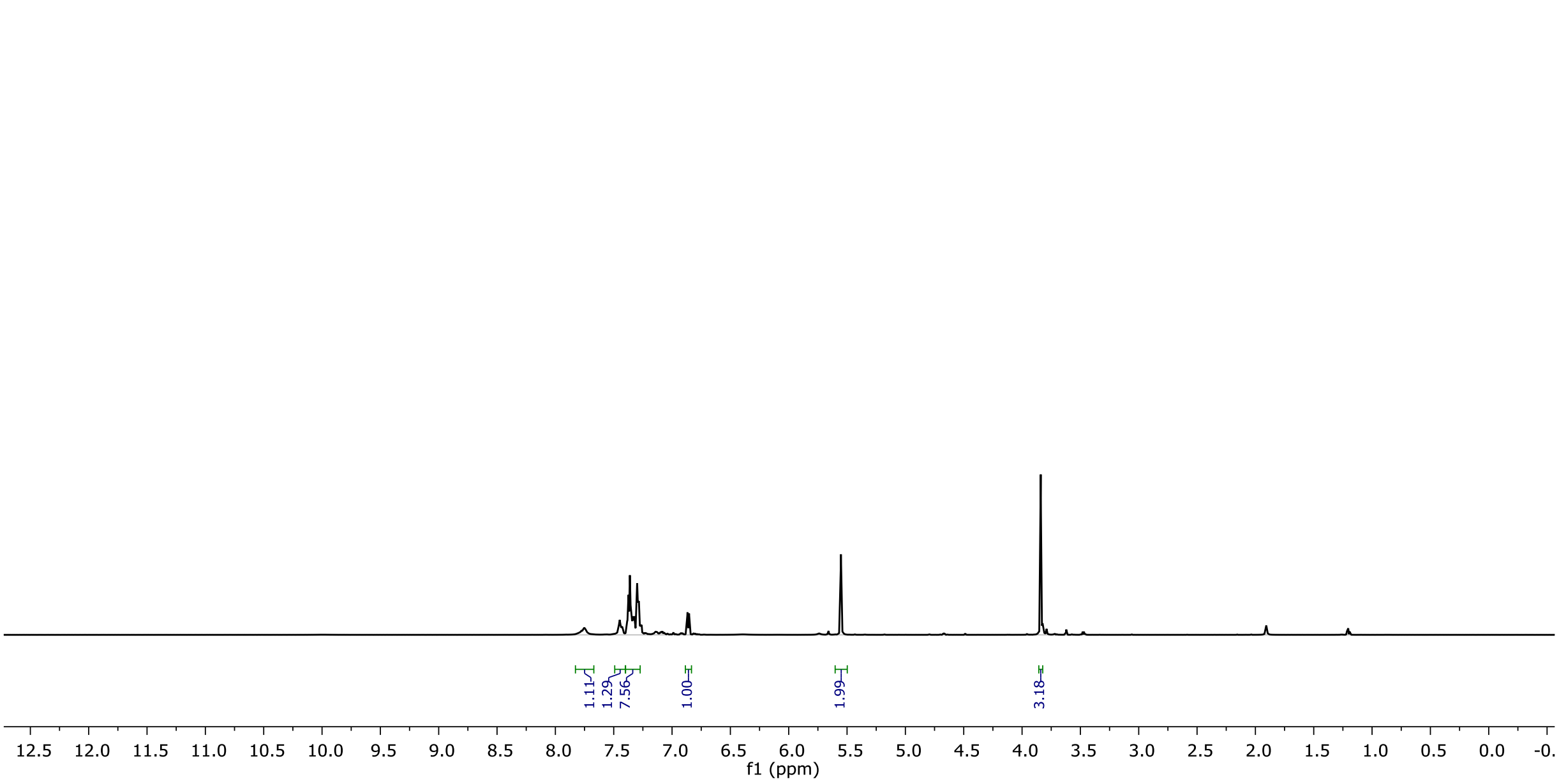

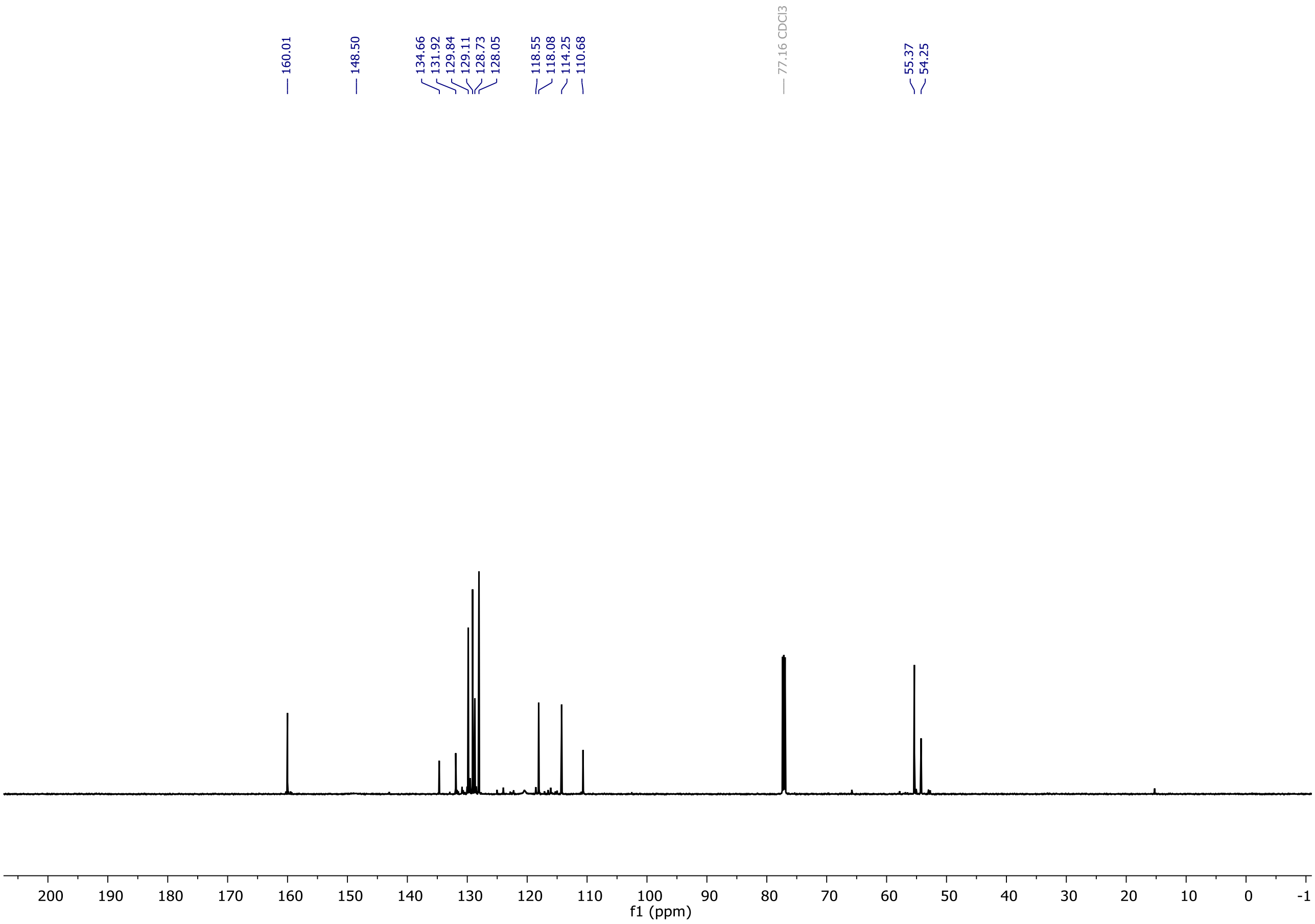

^{1}H and ^{13}C NMR spectra of compound **8**

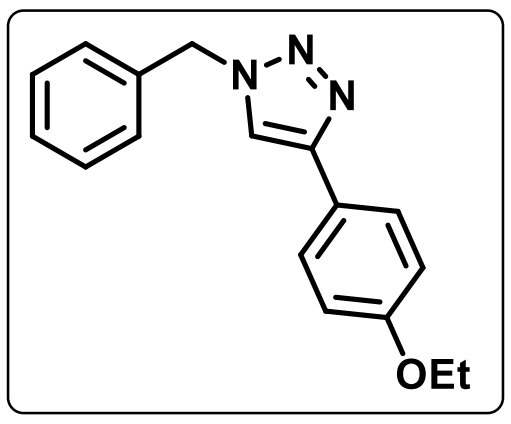

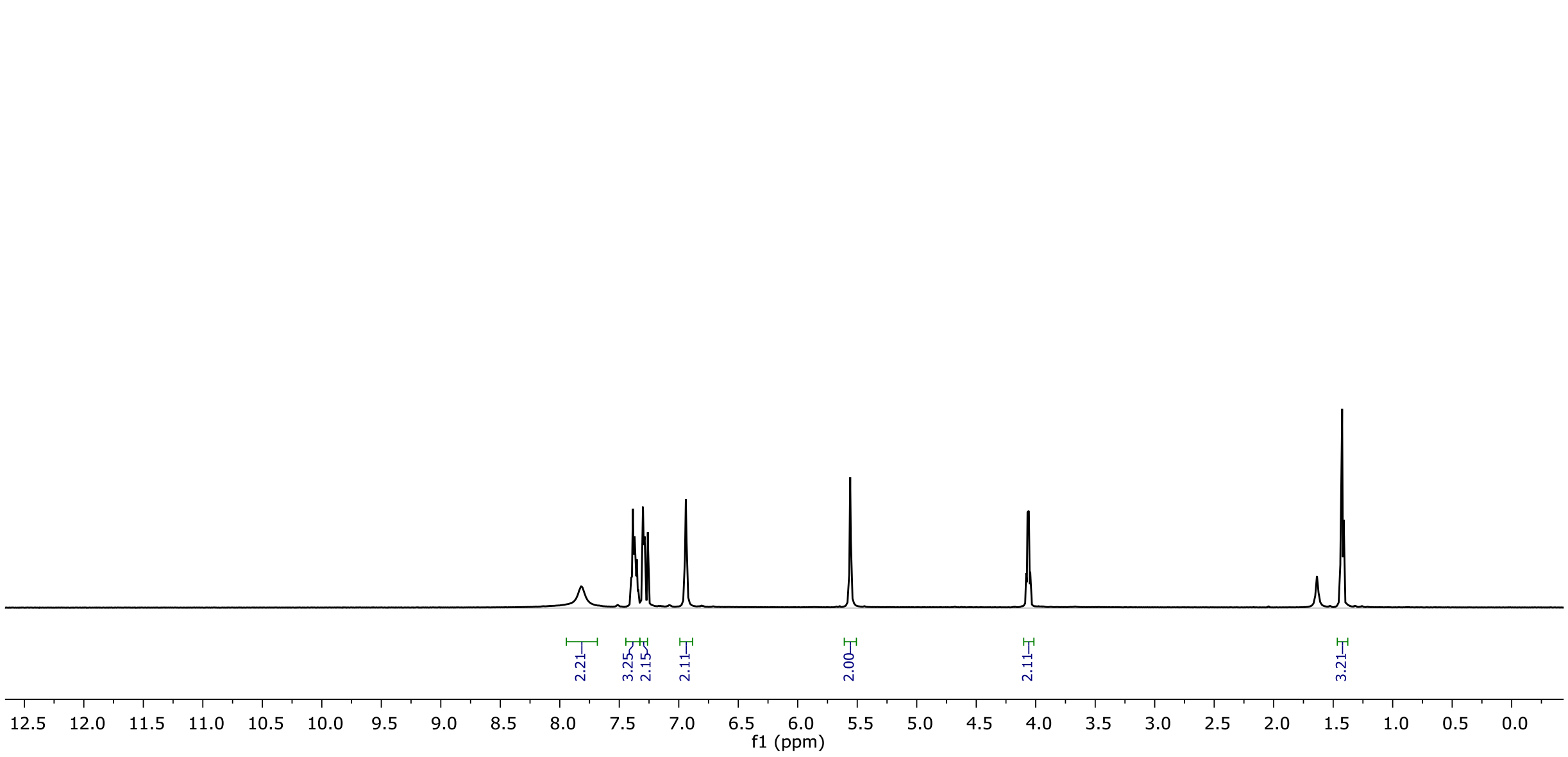

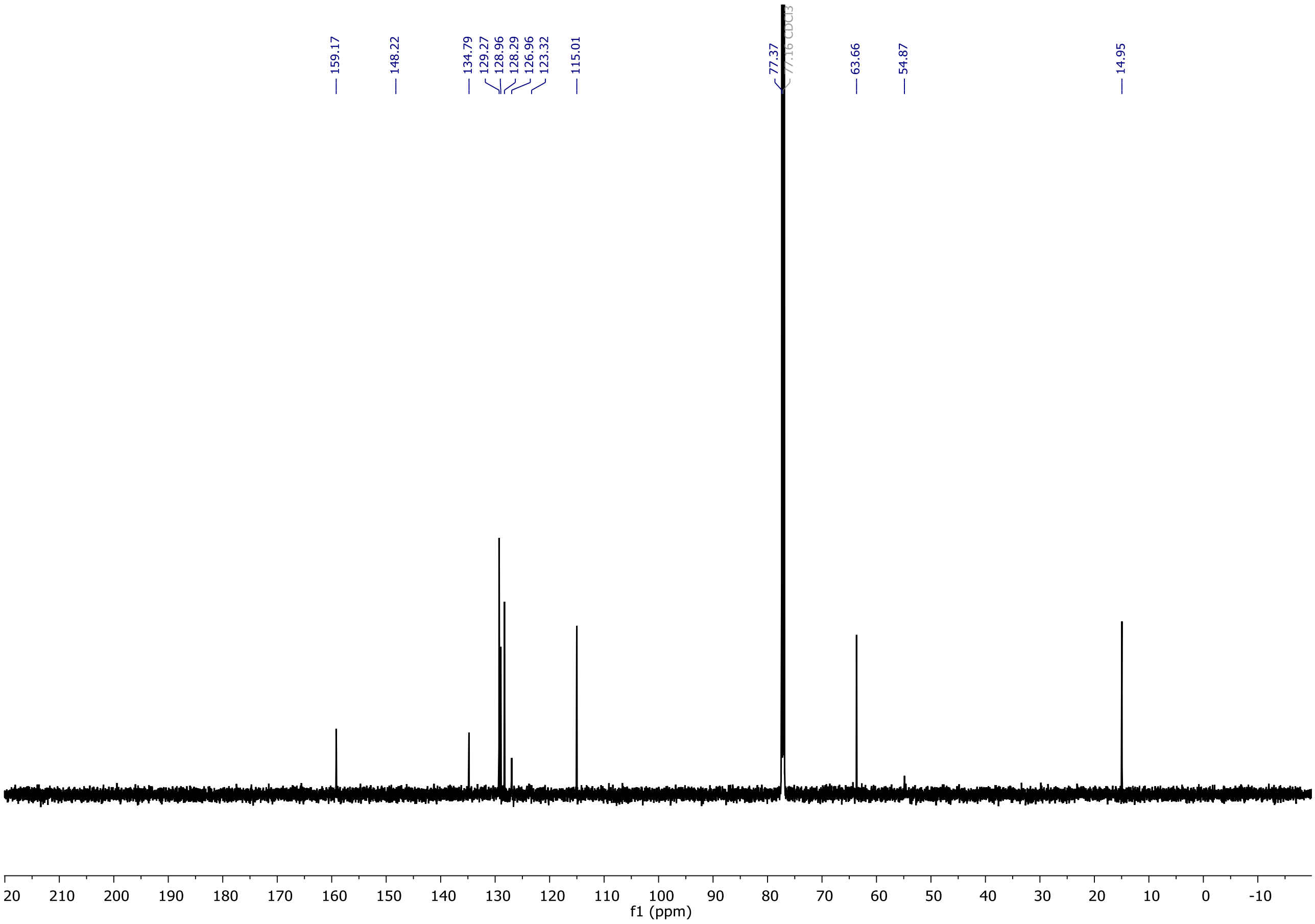

^{1}H and ^{13}C NMR spectra of compound **10**

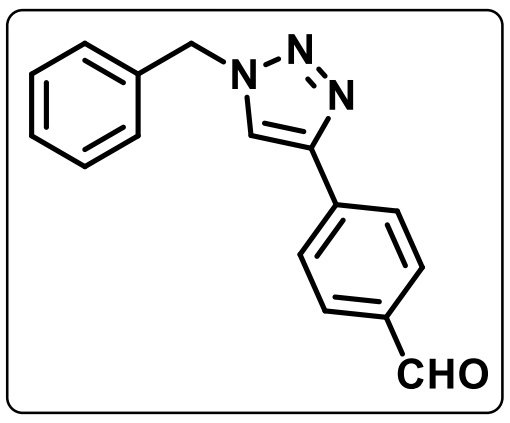

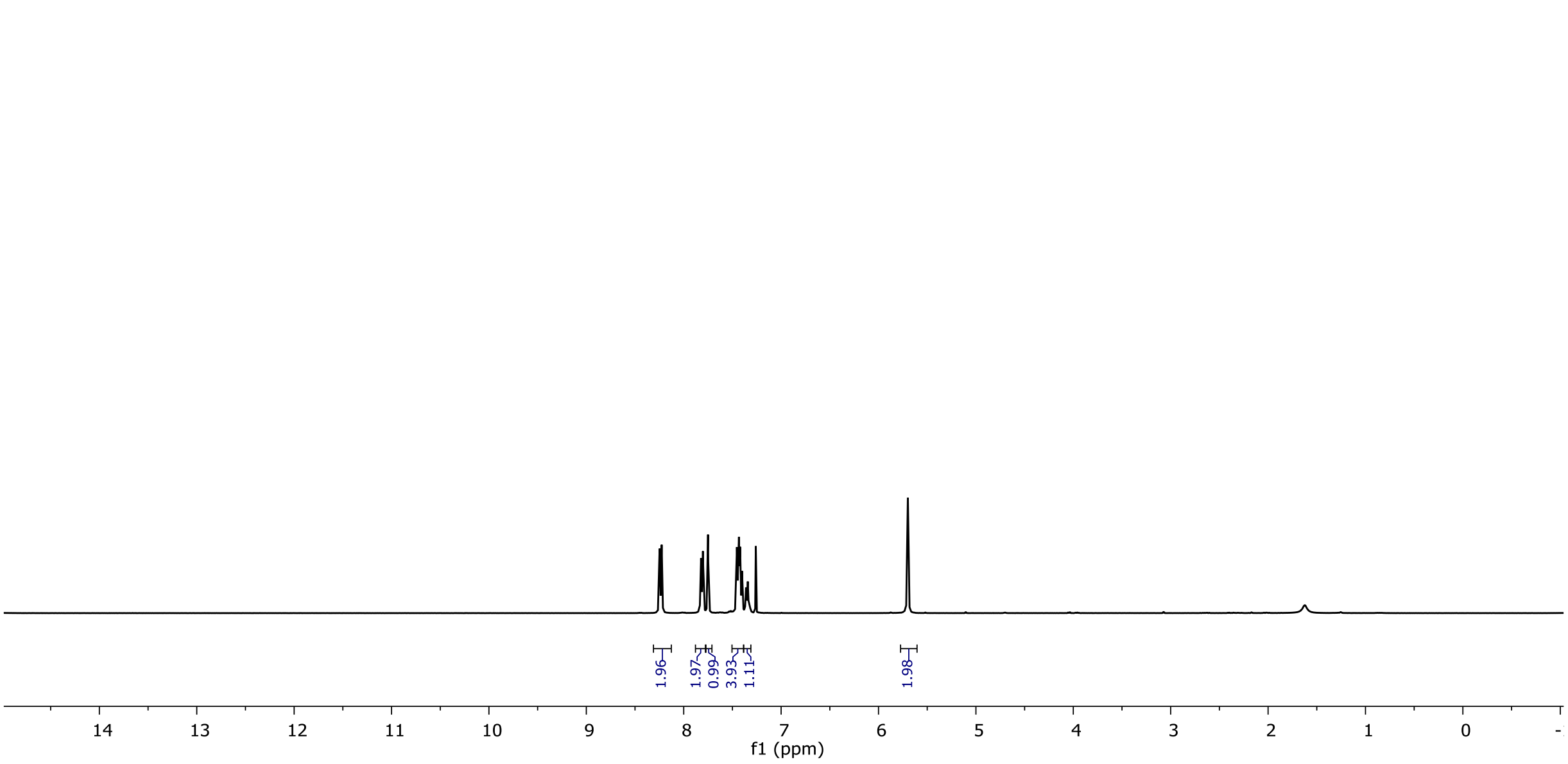

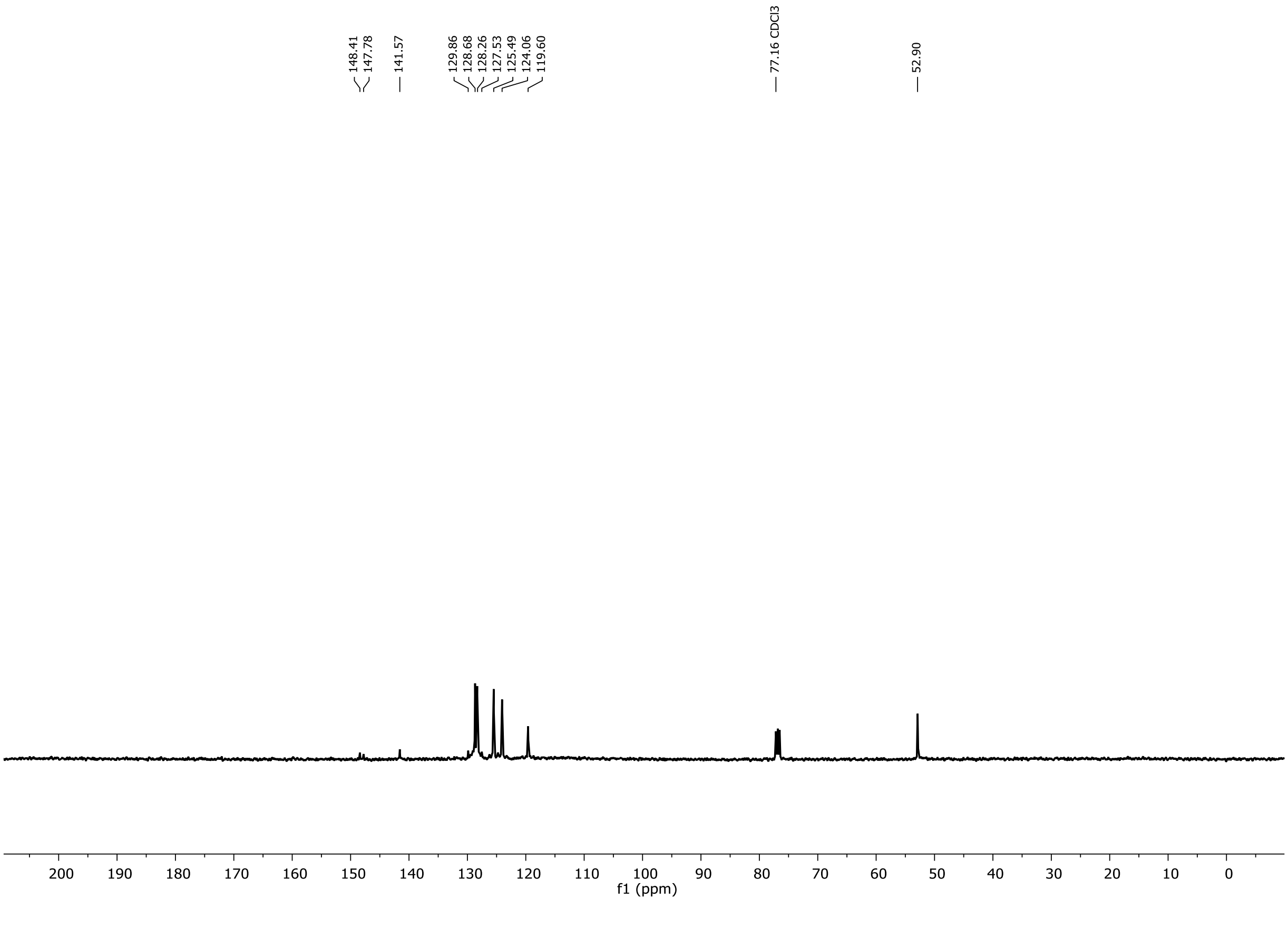

^{1}H and ^{13}C NMR spectra of compound **11**

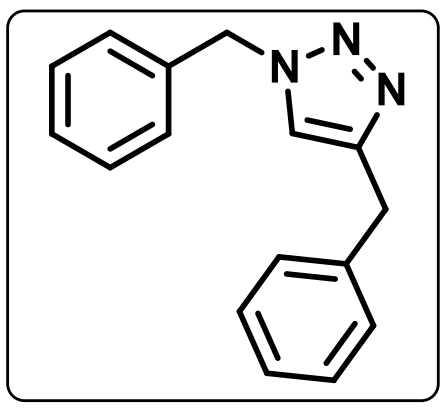

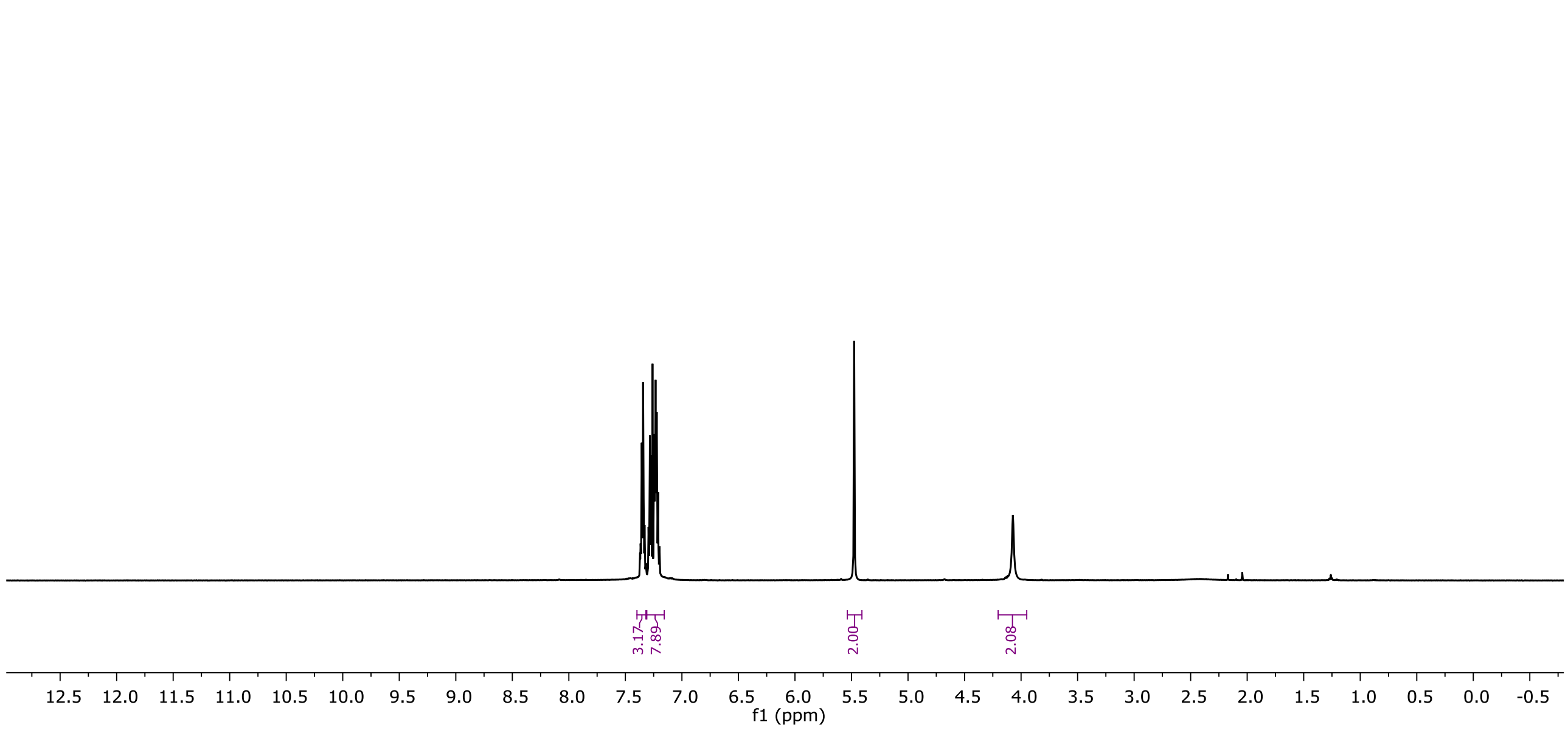

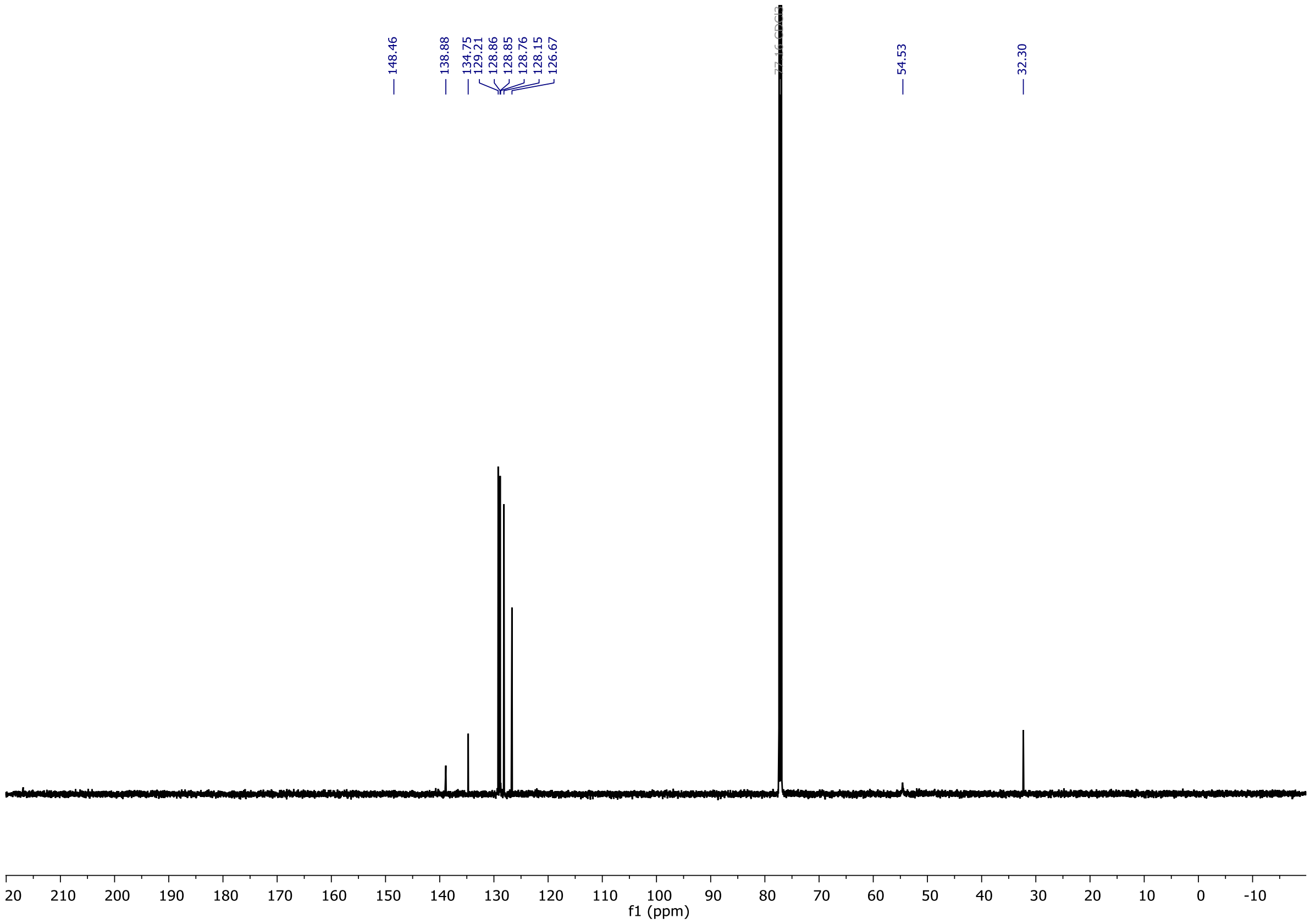

^{1}H and ^{13}C NMR spectra of compound **12**

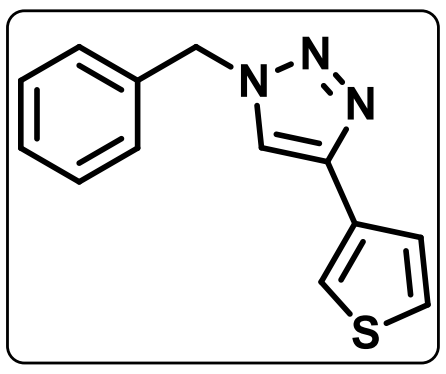

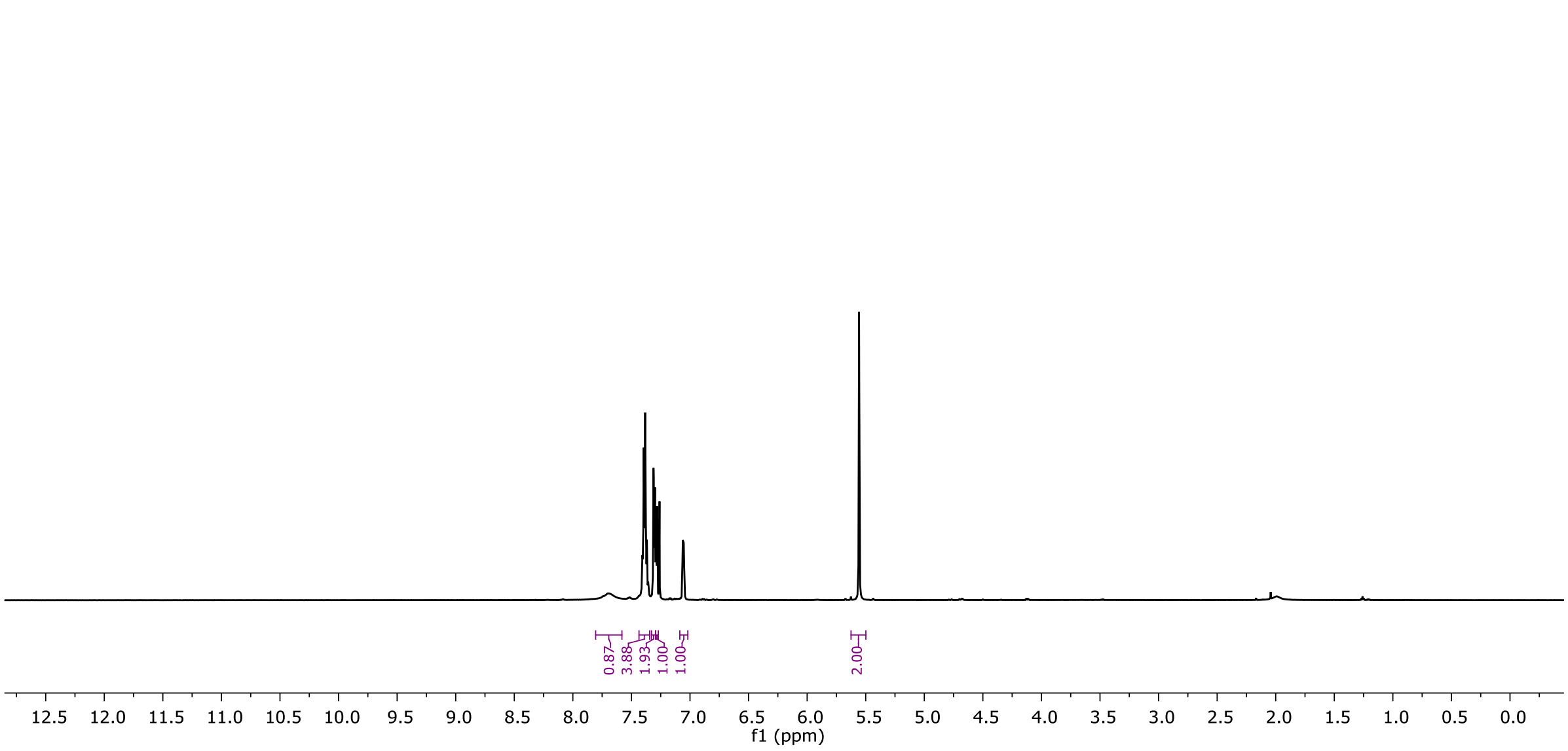

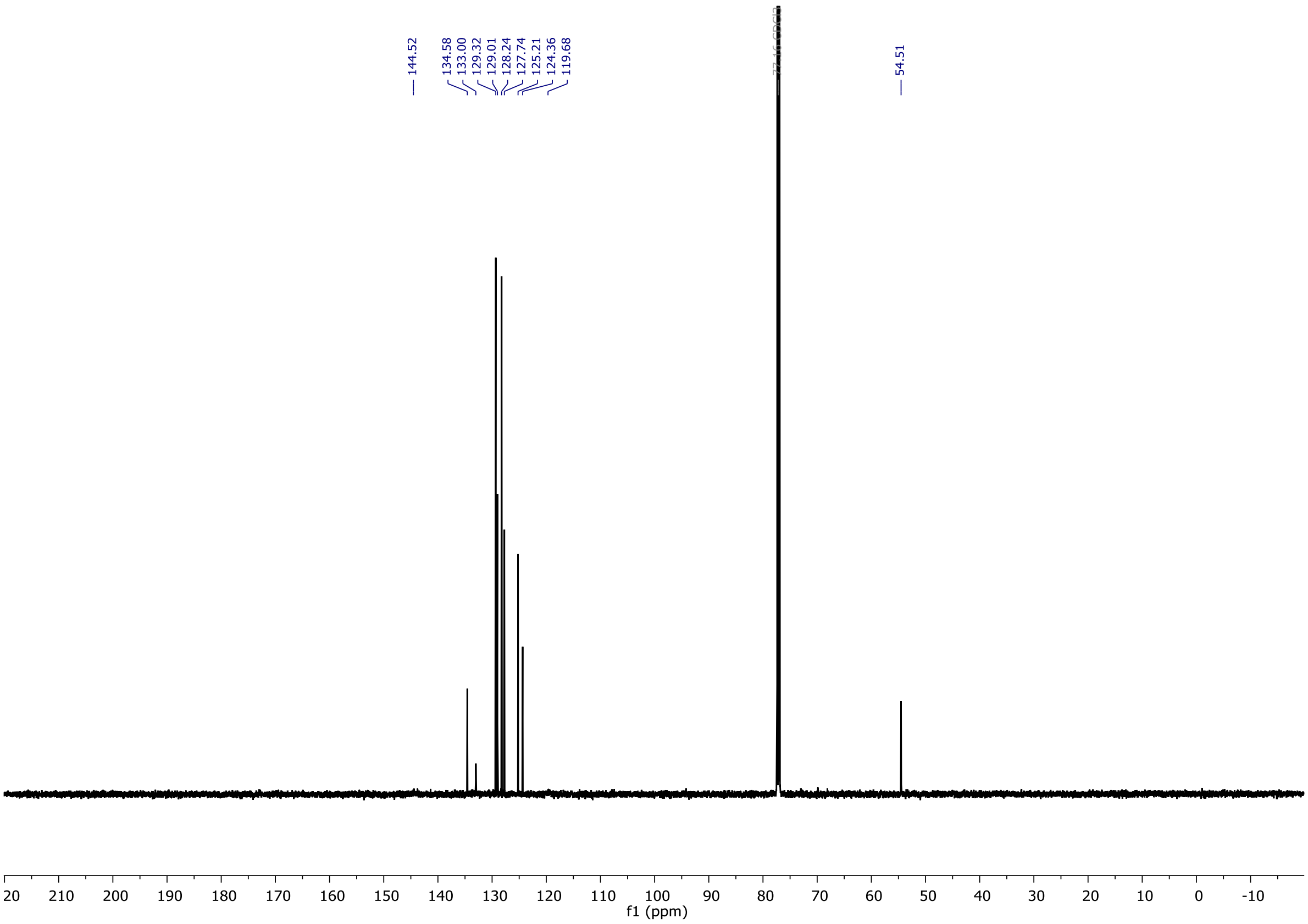

^{1}H and ^{13}C NMR spectra of compound **13**

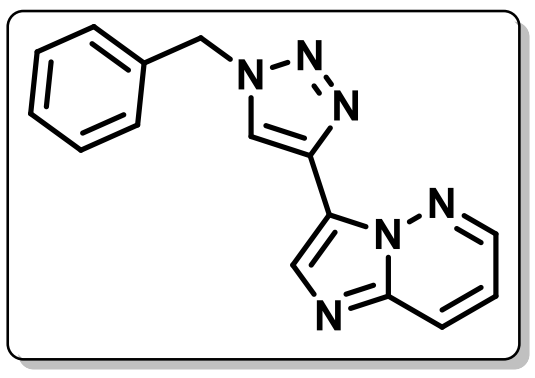

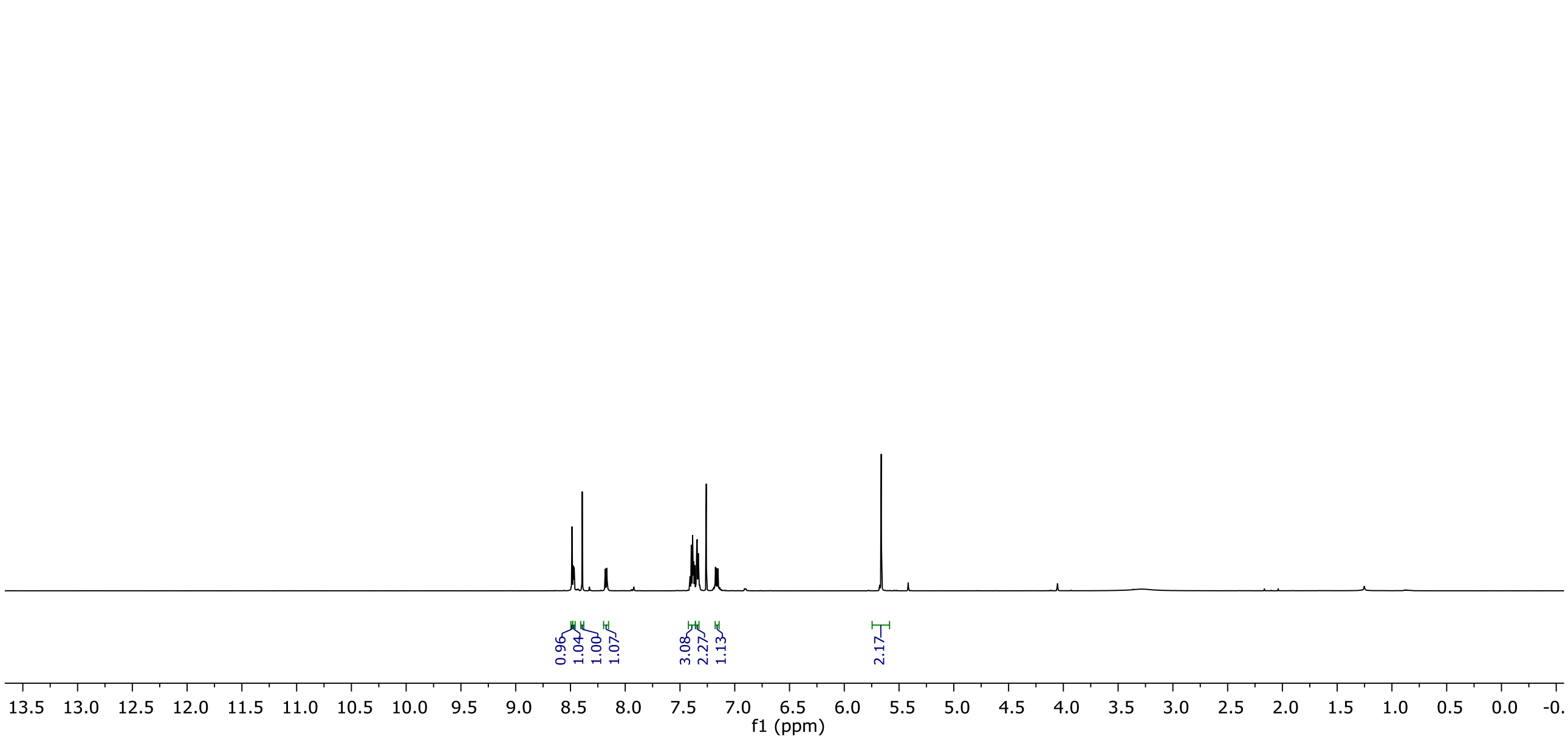

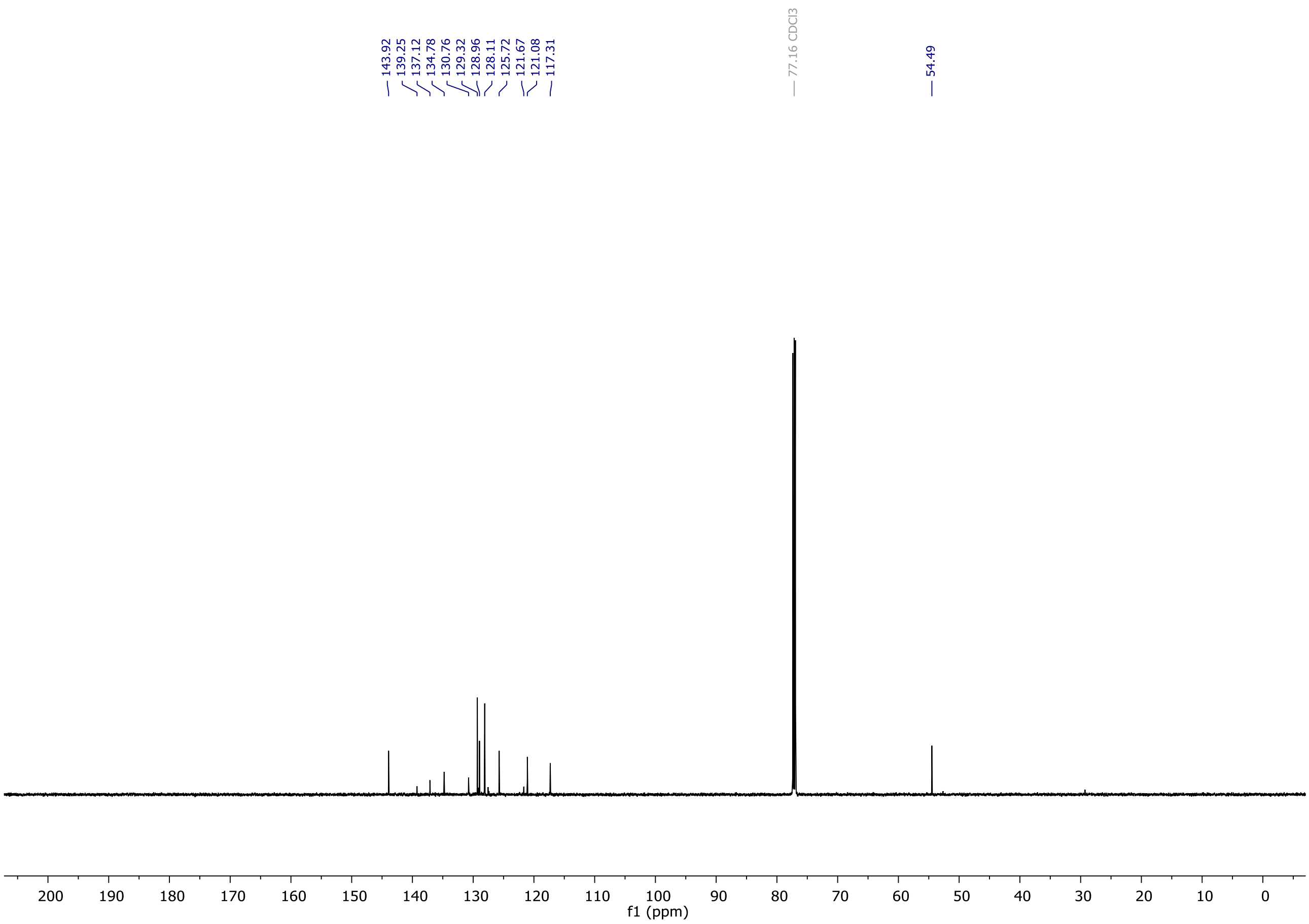

^{1}H and ^{13}C NMR spectra of compound **14**

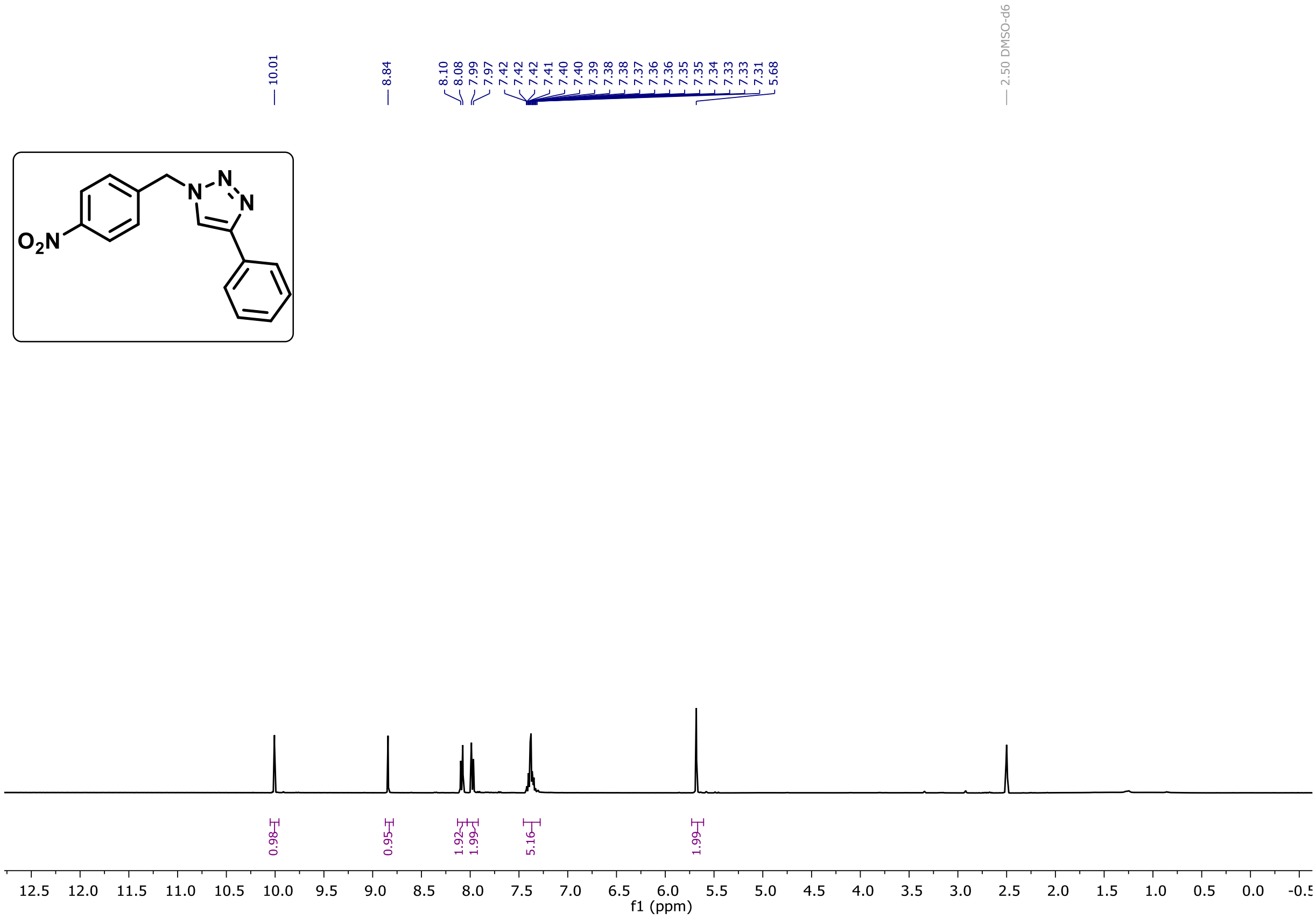

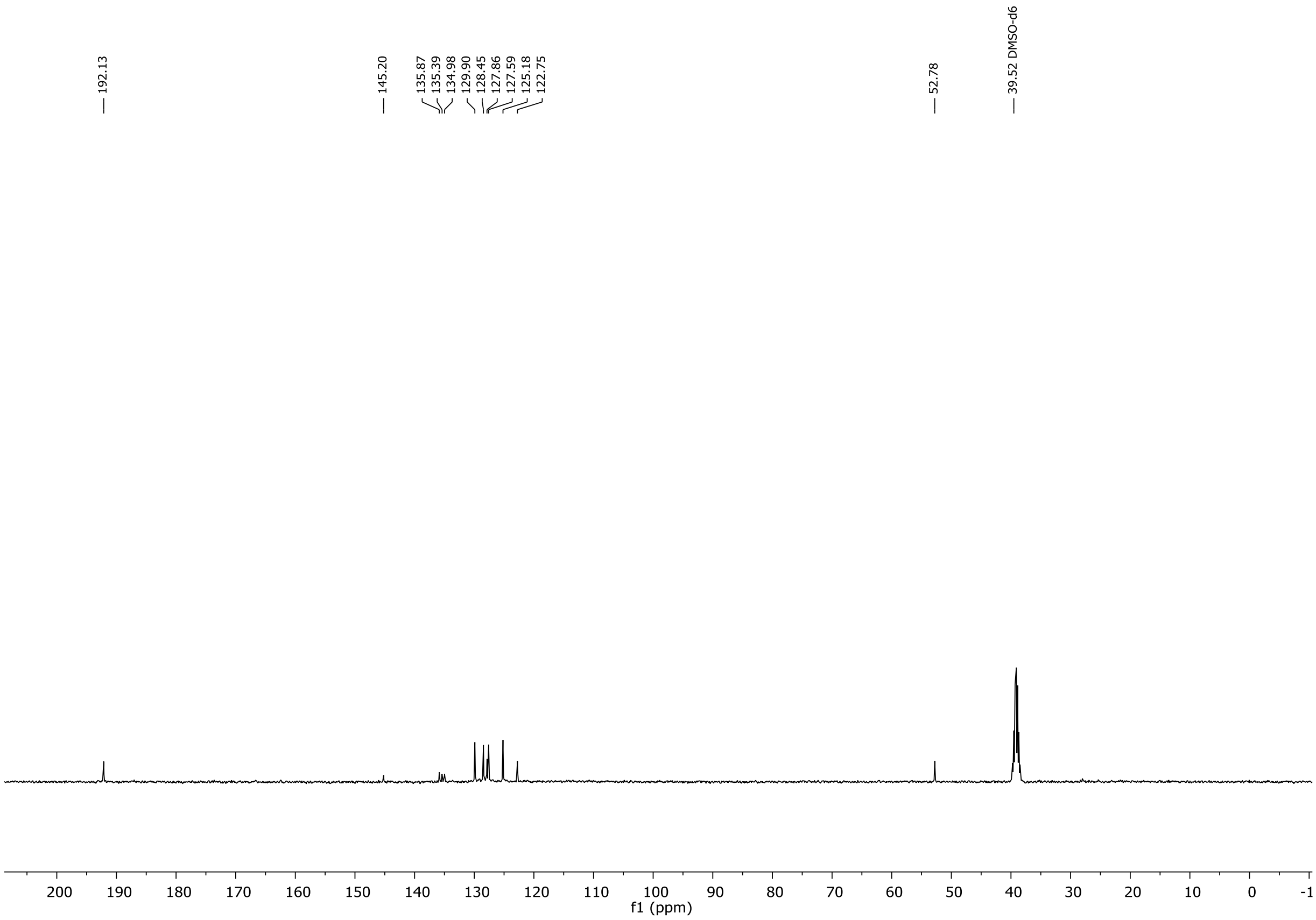

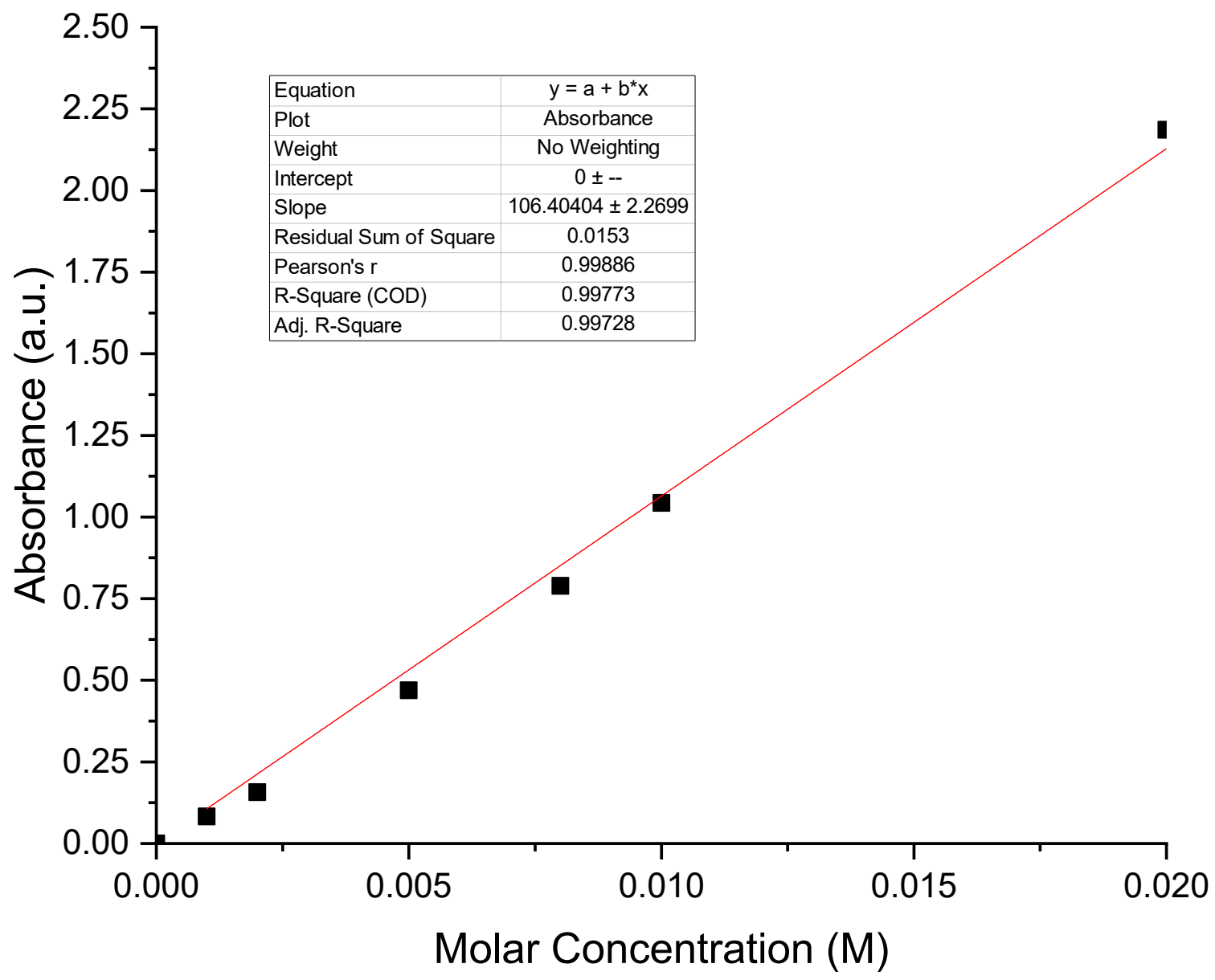

Figure S1. UV-Visiblecalibration curve obtained from standard $CuSO_4$ solutions of known concentration. The absorbance values were measured at λ = 264 nm and used to determine the residual Cu(II) concentration in solution after contact with the carbonate supports by linear regression analysis (R^2 = 0.9977).

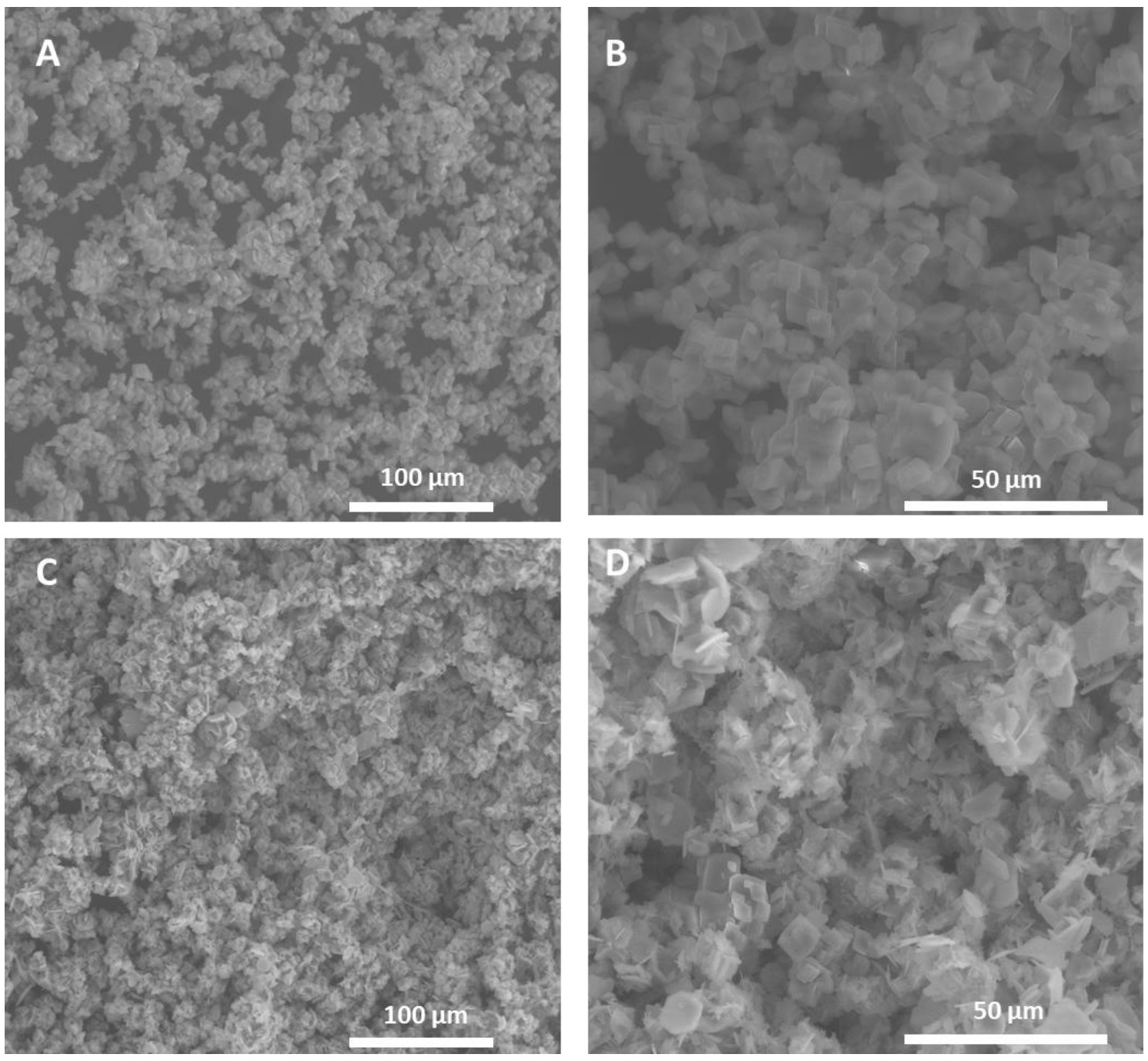

Element	Weights%	Atomics%
C K	16.0	25.7
O K	46.9	56.5
Al K	0.1	0.08
Ca K	36.6	17.6
Cu K	0.3	0.1
Totals	100	100

Element	Weights%	Atomics%
C K	9.2	16.4
O K	47.4	62.9
Al K	0.5	0.40
Ca K	7.7	5.1
Cu K	17.3	9.2
Totals	100	100

Figure S2. SEM analysis of $CaCO_3$ (A-B) and $CaCO_3$-Cu (C-D) at different magnifications with the corresponding EDX elemental analysis.

7. References

1. Mev, N.; Jain, Y.; Yadav, L.; Sharma, P.; Agarwal, M.; Gupta, R., Sonochemically synthesized copper chelate from expired streptomycin sulfate as a sustainable catalyst for green synthesis of 1,4-disubstituted triazoles in aqueous medium. *Tetrahedron* **2025,** *177*, 134564.
2. Taheri, P.; Molaei, P.; Abazari, A.; Tajbakhsh, M.; Yeganeh-Salman, E.; ghorbanian, M., Unveiling the synergistic interplay between Cu and Ni with ZIF-8 nanomaterials: a catalyst for the synthesis of triazole derivatives in nanoscale chemistry. *Res. Chem. Intermed.* **2025,** *51* (3), 1389-1415.
3. Song, Y.; Lee, S.; Dutta, P.; Ryu, J.-S., A Copper(I)-Mediated Tandem Three-Component Synthesis of 5-Allyl-1,2,3-triazoles. *Synthesis* **2020,** *52* (05), 744-754.
4. Sawkmie, M. L.; Paul, D.; Kalita, G.; Agarwala, K.; Maji, P. K.; Chatterjee, P. N., Synthesis and characterization of active cuprous oxide particles and their catalytic application in 1,2,3-triazole synthesis via alkyne-azide cycloaddition reaction in water. *J. Heterocyclic Chem.* **2019,** *56* (12), 3277-3288.
5. Saha, S.; Biswas, K.; Basu, B., 1-D copper(I) coordination polymer based on bidentate 1,3-dithioether ligand: Novel catalyst for azide-alkyne-cycloaddition (AAC) reaction. *Tetrahedron Lett.* **2018,** *59* (26), 2541-2545.
6. Chesnokov, G. A.; Topchiy, M. A.; Dzhevakov, P. B.; Gribanov, P. S.; Tukov, A. A.; Khrustalev, V. N.; Asachenko, A. F.; Nechaev, M. S., Eight-membered-ring diaminocarbenes bearing naphthalene moiety in the backbone: DFT studies, synthesis of amidinium salts, generation of free carbene, metal complexes, and solvent-free copper catalyzed azide–alkyne cycloaddition (CuAAC) reaction. *Dalton Trans.* **2017,** *46* (13), 4331-4345.
7. Krishnan, M.; Kathiresan, M.; Praveen, C., Electrochemically Generated Copper(I)-Catalyzed Click Chemistry: Triazole Synthesis and Insights into Their Photophysical Properties. *Eur. J. Org. Chem.* **2023,** *26* (12), e202201405.
8. Harmand, L.; Lescure, M.-H.; Candelon, N.; Duttine, M.; Lastécouères, D.; Vincent, J.-M., Huisgen click cycloadditions from a copper(II)-tren precatalyst without external sacrificial reductant. *Tetrahedron Lett.* **2012,** *53* (11), 1417-1420.
9. Riethmann, M.; Föhrenbacher, S. A.; Keiling, H.; Ignat'ev, N. V.; Finze, M.; Radius, U., Fluoride Abstraction Induced by Tris(pentafluoroethyl)difluorophosphorane: A Convenient Way to Synthesize Cationic N-Heterocyclic Carbene- and Cyclic (Alkyl)(amino)carbene-Ligated Copper Alkyne and Arene Complexes. *Inorg. Chem.* **2024,** *63* (18), 8351-8365.
10. Liu, G. F.; Li, Z. W.; Huang, Z. J.; Zhou, Z.; Li, Y. X.; Huang, A.; Cai, Z.; Ouyang, G.; Ye, B. H.; Zhang, Y. B., Rapid Crystallization and Versatile Metalation of Acetylhydrazone-Linked Covalent Organic Frameworks for Heterogenous Catalysis. *J. Am. Chem. Soc.* **2025,** *147* (2), 1840-1850.
